# Advances of Green‐Synthesized Nanoparticles for Biomedical Applications

**DOI:** 10.1155/bmri/9207147

**Published:** 2026-04-02

**Authors:** Md Hosne Mobarak, Md. Zobair Al Mahmud, Amran Hossain, Md. Arman Hossain Abir, Nayem Hossain, Mohammad Asaduzzaman Chowdhury

**Affiliations:** ^1^ Department of Mechanical Engineering, IUBAT-International University of Business Agriculture and Technology, Dhaka, Bangladesh, iubat.edu; ^2^ Department of Mechanical Engineering, Dhaka University of Engineering and Technology (DUET), Gazipur, Bangladesh, duet.ac.bd

**Keywords:** characterization, nanoparticles, pharmaceutical, plants, synthesis

## Abstract

In biomedical nanotechnology, plant‐mediated production of metallic nanoparticles has become a viable and physiologically adaptable method that offers decreased toxicity, enhanced biocompatibility, and functional surface modification by phytochemical capping. In addition to altering physicochemical characteristics, including size, shape, crystallinity, and surface charge, plant extracts also function as reducing, stabilizing, and capping agents, allowing for controlled nanoparticle production. Antimicrobial efficacy, cytotoxic selectivity, antioxidant activity, and interactions with mammalian cells are among the properties that significantly influence biological performance. The mechanistic knowledge of nanoparticle production and structure–activity correlations has been made easier by developments in spectroscopic, microscopic, and surface analytical methods. When taken as a whole, plant‐derived nanoparticles show encouraging biomedical potential in antimicrobial therapy, anticancer applications, wound healing, and drug delivery assisted by nanocarriers. However, they also pose issues with standardization, reproducibility, and translational scalability. This semisystematic review summarizes recent advancements in the synthesis, characterization, and biomedical applications of plant‐derived nanoparticles, highlighting quantitative trends, mechanistic insights, and important knowledge gaps pertinent to future clinical translation. It covers literature from 2015 to 2024 and analyzes over 120 studies.

## 1. Introduction

Since the discovery of nanomaterials, research focusing on materials with diameters of 100 nm or less has expanded rapidly across multiple scientific disciplines [1]. Nanoparticles (NPs) have demonstrated different catalytic, thermal, optical, electrical, and biological capabilities used in various fields due to their high surface energy, large surface area to volume ratio, and relatively small size compared to bulk material [2]. Compared to traditional chemical and physical synthesis methods, plant‐mediated green synthesis of NPs is a sustainable, cost‐efficient, and scalable form that does not produce harmful side products and severe reaction conditions [3]. In this review, attention is drawn to the synthesis of NPs using plant‐derived biomolecules, but not microbial or enzymatic pathways.

In the current years, there has been an upsurge in the number of research directed toward sustainable NP synthesis, specifically, through the use of plants. Unlike the conventional chemical and physical methods that usually require toxic reagents and energy‐dense methods in order to complete the processes, green synthesis utilizes the inherent phytochemicals in eliminating and stabilizing metallic ions at ambient conditions. The methodological path follows the green chemistry principles and favors biomedical innovation through providing biocompatible, environmentally friendly, and economically accessible options [4, 5]. According to Hano and Abbasi, plant‐mediated synthesis has gained significant momentum due to its broad applicability and flexibility in drug delivery, tissue engineering, and antibacterial therapeutics [6]. Although this has increased, there are still inconsistencies in the standardization of plant species, task preparations, and synthesis procedures. This review answers these questions by evaluating the advances and deficiencies of plant‐based green synthesis methods over the years 2015–2024 in a critical analysis.

A crucial step in creating plant‐derived NPs is using the naturally occurring reducing and stabilizing compounds present in various plant components, including leaves, stems, roots, and fruits [4]. This strategy, also called “green synthesis,” offers a viable and environmentally responsible replacement for traditional chemical synthesis techniques [7]. Green synthesis techniques lessen the need for dangerous chemicals and provide a scalable, affordable method for producing NPs [6].

Understanding the physicochemical characteristics of plant‐derived NPs and confirming their suitability for particular applications require thorough characterization [8]. These NPs can be characterized at the nanoscale using cutting‐edge analytical techniques like spectroscopy, microscopy, and elemental analysis, which reveal their size, morphology, composition, surface properties, and stability. Such characterization enables the customization of plant‐based NP properties to meet the needs of various applications [9].

Due to their extraordinary potential for drug delivery and therapeutic applications, plant‐derived NPs have attracted considerable interest in the pharmaceutical industry [10]. These NPs have several benefits, such as controlled drug release, targeted delivery, and biocompatibility [11]. Plant‐derived NPs have found use in dental, metal, and ceramic implants, providing unique benefits in improved biocompatibility and performance [12]. These NPs can be added to improve the mechanical qualities of implant materials, such as strength and durability [13].

In spite of these benefits, green synthesis via plants faces some challenges related to their inability to be easily repurchased over time, difficulty in increasing the manufacturing volume, and absence of standard procedures, which make it increasingly hard to gain regulatory clearance and translate into medicine. This review is aimed at giving a thorough overview of the synthesis, characterization, and applications of plant‐derived NPs. We hope that by highlighting these NPs′ unique qualities and various uses, we can encourage more investigation into and advancements in this quickly developing area. The study of environmentally friendly and nature‐inspired methods in nanotechnology shows great promise for tackling significant societal problems and opening up new horizons for cutting‐edge technologies.

This semisystematic review analyzed literature from January 2015 to February 2024 using databases such as Scopus, Web of Science, PubMed, ScienceDirect, and Google Scholar. Search terms included combinations of “green synthesis,” “plant extract,” “nanoparticles,” “silver nanoparticles,” “ZnO nanoparticles,” and “biomedical applications” using Boolean operators. Articles were included if they were peer‐reviewed, in English, and involved plant‐mediated synthesis of NPs with at least one characterization technique and biomedical relevance. Excluded were reviews, non‐English texts, microbial/enzymatic synthesis studies, or those lacking experimental detail. From an initial pool of around 230 articles, 120 were selected after full‐text screening. While studies often demonstrated promising results, reproducibility remains a significant issue due to inconsistent synthesis parameters—such as plant species, extract preparation methods, pH, and temperature—leading to variability in NP size, yield, and stability. For example, silver nanoparticles (AgNPs) synthesized using *Azadirachta indica* frequently showed consistent outcomes, whereas other NPs, like ZnO and gold nanoparticles (AuNPs), exhibited wider variability. These findings highlight the need for standardized green synthesis protocols and detailed phytochemical profiling to enhance reproducibility and facilitate clinical translation.

## 2. Methodology of Review

Bionanotechnology has significantly changed contemporary science and technology [14]. The appeal of NPs, particularly those made of precious metals, lies in their minute size and diverse applications in various scientific fields that serve the greater good of humanity [15]. Utilizing plants to create metal NPs is advantageous as it is ecologically sound, cost‐effective, and simplifies the synthesis process [16]. The application of nanotechnology has emerged as a significantly promising technology [17]. Nanotechnology creates metal NPs, and biomedical technology′s widespread use has gained worldwide recognition due to its versatile applications [18]. Recently, the production of metal NPs by synthesis has been on the rise [19]. In‐depth research has been conducted on utilizing microorganisms and plants, which has been acknowledged as an intelligent and ecofriendly approach for utilizing microorganisms as functional nanofactories [20].

Using dried plant or algae extracts is a general approach for the ecofriendly production of metal NPs [21]. Biomolecules with unique properties that are derived from plants, algae, and microorganisms, known as natural pigments, have been utilized in the biological synthesis of NPs [22]. The research on the bioreduction of NPs using dyes has been comparatively limited [23]. Nevertheless, it has been acknowledged that pigments exhibit a robust capacity for reducing and stabilizing properties during NP synthesis through biological manufacturing [24]. Currently, a wide range of plants and their byproducts are available, and several botanical insecticides have shown remarkable effectiveness and have been made available for commercial use [25]. With the advancement of science and technology, scientists are now creating herbal drugs in nanoform to improve their research [26]. At this moment, an intelligent solution could be to utilize plants and their derivatives in a nanosized form to combat the increasing population of larvae effectively [27].

**Figure 1:**
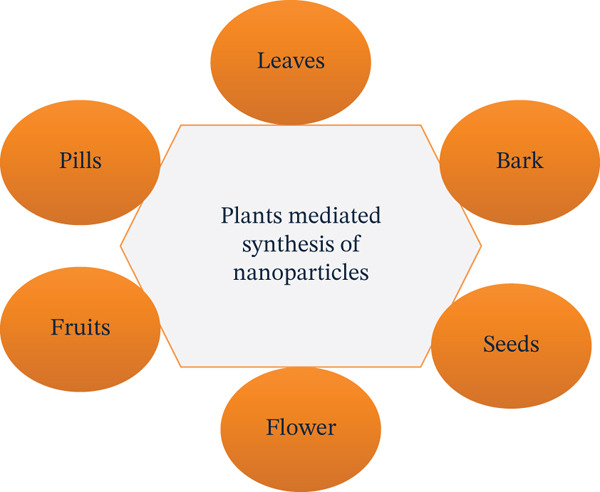
The synthesis of nanoparticles involves using various components of plants [28].

In many different sectors and areas, it is common practice to use different plant parts, such as fruits, stems, bark, seeds, latex, and callus, as illustrated in Figure 1. The aforementioned material is used to create NPs by biosynthesis [29–33]. The transition of color from light green to dark brown indicates that plants can create NPs quite quickly compared to other systems [34]. A slight change in the pH and temperature of the NPs can modify their shape and size [35, 36].

**Figure 2:**
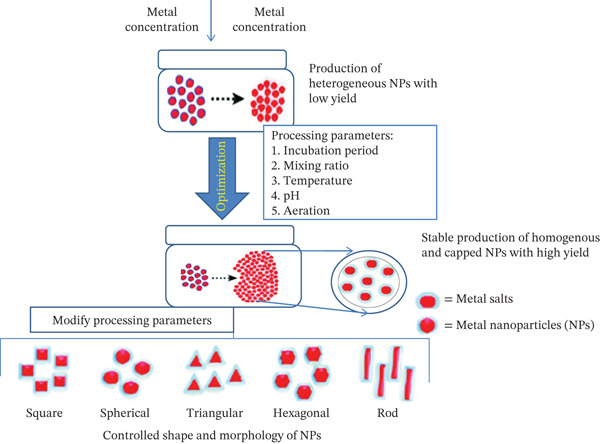
The biosynthesis of metallic nanoparticles entails a series of stages [37].

Figure 2 illustrates the optimization process in the green synthesis of NPs. Initially, heterogeneous NPs form with low yield. By adjusting parameters like incubation time, mixing ratio, temperature, pH, and aeration, the process yields stable, homogeneous, capped NPs. Shape and morphology—square, spherical, triangular, hexagonal, or rod—can be controlled by modifying processing conditions. It is well‐established that diverse biological organisms produce NPs with distinct physical characteristics [38]. Utilizing microbial cells′ highly organized physical and biological activity is a novel technique that has recently come to light for producing metal NPs [39].

## 3. Plant‐Based Green Synthesis

Through various methods, including those that utilize physical and chemical strategies (known as the top–down approach), as well as biological and chemical processes (known as the bottom–up method), researchers have succeeded in synthesizing NPs [40]. Green synthesis, utilizing natural methods, has been discovered to be an environmentally responsible and less hazardous alternative to physical and chemical techniques, contributing to contamination and toxicity [41].

Methods for synthesizing AgNPs are shown in Figure 3. Figure 3a demonstrates how the form and size of NPs are influenced by top–down (mechanical slicing) and bottom–up (atomic reduction, growth, and capping) techniques. Figure 3b divides synthesis methods into three categories: chemical (sol–gel, pyrolysis), biological (plants or microorganisms like bacteria, fungi, and algae), and physical (ball milling).

Figure 3(a) Top–down and bottom–up approaches and (b) various methods of synthesis of nanoparticles [42].

**Figure:**
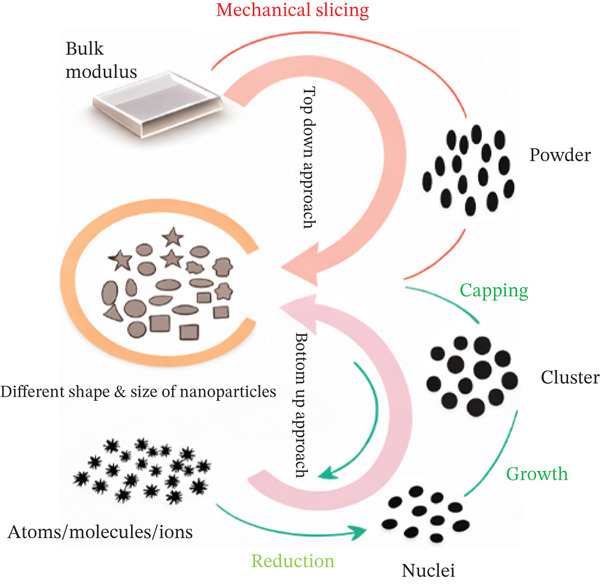
(a)

**Figure:**
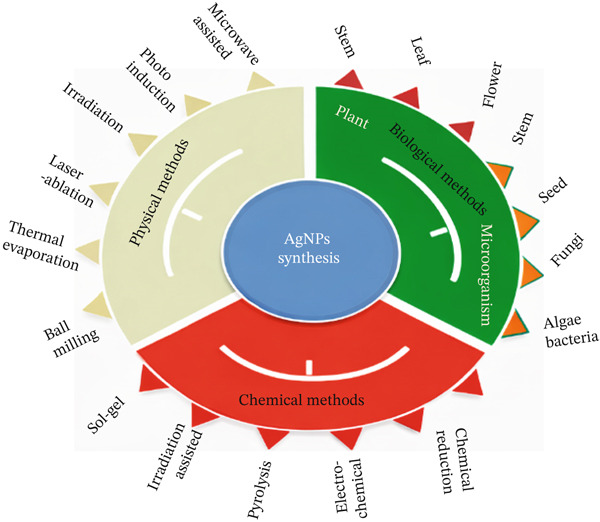
(b)

The production of nanomaterials can be categorized based on the approach employed, namely, the bottom–up and top–down methods [43]. According to the “top–down” method, large materials or particles are gradually broken down into smaller ones using deliberate and organized physical processes like crushing, milling, grinding, lithography, and etching techniques like ion and plasma etching [44]. The primary limitations associated with this approach include elevated energy consumption levels, an incapacity to synthesize particles of a smaller size, and an imperfect surface morphology that could potentially engender significant impacts on the physical and surface features of nanomaterials [45]. The bottom–up approach entails creating NPs of various sizes and shapes by arranging the atoms and molecules of a substance [46]. This technique frequently creates nanomaterials with predictable dimensions, structures, and dispersion [47]. Numerous methods, such as plant‐mediated green synthesis, chemical vapor deposition (CVD), sol–gel synthesis, laser pyrolysis, microwave heating, self‐assembly of monomer/polymer molecules, and supercritical hydrothermal processing, are widely used in the bottom–up approach. Following its distinct qualities, synthesis can be separated into three categories: physical, chemical, and biological processes [48].

**Figure 4:**
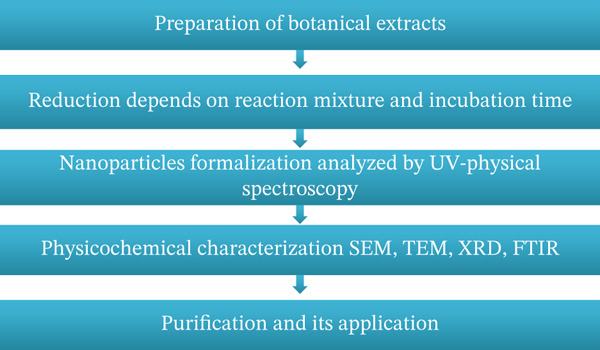
Steps included within the biosynthesis of nanoparticles [53].

The green synthesis methodology presents a promising approach, which entails using natural compounds to reduce, cap, and stabilize agents, thereby replacing the utilization of costly and hazardous chemical substances [49]. Numerous biological resources, including distinct plant components (roots, leaves, fruit, etc.), bacteria, fungi, and algae, among others, exist. The method above offers the possibility of environmentally friendly NP manufacturing that is biologically active [50–52]. The green production of NPs utilizing botanical extracts is shown in Figure 4. Extract preparation; bioreduction impacted by incubation conditions; UV‐vis spectroscopy analysis of NP creation; physicochemical characterization using scanning electron microscope (SEM), transmission electron microscopy (TEM), x‐ray diffraction (XRD), and Fourier transform infrared (FTIR); and purification and application of the generated NPs are all included.

The most effective plants for synthesizing NP are those with bioaccumulation and heavy metal detoxifying abilities [54]. Plant extract biomolecules have the potential to function as organic capping and reducing agents during the green synthesis of NPs. The types and portions of the plant, as well as the extraction process, affect the content of these metabolites [55].

From Table 1, it is evident that some plant extracts are more effective than others in producing smaller or more stable NPs. For instance, banana peel extract yielded cadmium sulfide NPs as small as 1.48 nm, while *Sargassum* alga produced palladium NPs averaging 5–10 nm. In contrast, *Cocos nucifera* leaf extract resulted in relatively larger lead NPs around 47 nm. These differences are likely due to the varying phytochemical compositions and reducing capacities of the plant extracts, which influence nucleation and growth mechanisms during synthesis. Such comparative data help in selecting optimal plant sources for desired NP characteristics.

**Table 1:** Plant‐based synthesis of nanoparticles.

Plants	Extracting plant tissues	Types of nanoparticle	Shapes	Size	Refs
*Euphorbia prostrata*	Leaves	Silver and titanium dioxide (TiO_2_)	Spherical	Silver, 10–15 (nm); TiO_2_, 81.7–84.7 (nm)	[56]
*Sargassum*	Alga	Palladium	Octahedral	5–10 (nm)	[57]
*Ginkgo biloba*	Leaves	Copper	Spherical	15–20 (nm)	[58]
Panax ginseng	Root	Silver and gold	Spherical	Silver, 10–30 (nm); gold, 10–40 (nm)	[59]
Red ginseng	Root	Silver	Spherical	10–30 (nm)	[60]
*Cocos nucifera*	Leaves	Lead	Spherical	47 (nm)	[61]
Banana	Peel	Cadmium sulfide	—	1.48 (nm)	[62]
*Citrus medica*	Fruit	Copper	—	20 (nm)	[63]
Orange and pineapple	Fruits	Silver	Spherical	10–300 (nm)	[64]
*Gardenia jasminoides*	Leaves	Iron	Rock‐like appearance	32 (nm)	[65]
*Azadirachta indica*	Leaves	Silver	—	41–60 (nm)	[66]
*Nigella sativa*	Leaves	Silver	Spherical	15 (nm)	[67]
*Catharanthus roseus*	Leaves	Palladium	Spherical	40 (nm)	[68]
*Pistacia atlantica*	Seeds	Silver	Spherical	27 (nm)	[69]
*Nyctanthes arbortristis*	Flower	Silver	—	—	[70]
*Artocarpus gomezianus*	Fruit	Zinc	Spherical	> 20 (nm)	[71]
*Cymbopogon citratus*	Leaves	Gold	Triangular, hexagonal, spherical, and rod	20–50 (nm)	[72]

### 3.1. Copper and Copper Oxide Nanoparticle (CuO NP) Synthesis Through Plant‐Based Green Method

Plants are rich in phytochemicals and secondary metabolites that can be utilized as valuable bioresources to synthesize Cu and CuO NPs [73, 74]. Phenols and flavonoids are the primary phytochemicals present in various parts of plants, like leaves, roots, shoots, stems, flowers, and fruits [75]. These phenols contain hydroxyl and ketone groups, which aid with iron chelation and ultimately exhibit potent antioxidant characteristics, according to [76]. Using this environmentally friendly approach, the stability of NPs was enhanced while also preventing their aggregation and distortion, creating an opportunity for the absorption of phytochemicals onto the NPs′ surface. This absorption process increased the NPs′ reaction rate [77]. Utilizing green synthesis techniques, as opposed to conventional chemical and physical techniques, produces CuO NPs in a way that is significantly more ecologically conscious and safe [78]. Researchers are currently studying the impact of CuO NPs, produced through environmentally friendly means, on the green peach aphid [79].

### 3.2. Green AgNP Synthesis Based on Plants

Extracts of *Achillea millefolium* were used to reduce and stabilize SNPs [80]. The plant was gathered at Rawalakot and given to the university′s herbarium there. Before being turned into powder, the plant was first washed and dried. To create plant extracts, 250 mL of distilled water, ethanol, and methanol were added to 25 g of powder and vigorously shaken for 24 h at room temperature. After filtering with Whatman Number 1 filter paper, the filtrate was used to reduce silver ions in the AgNO_3_ solution. The creation of SNPs was shown by the brown hue that resulted from the reaction of 20 mL of plant extract with 80 mL of AgNO_3_ (1 mM) solution over 24 h at room temperature. The morphology of SNPs was examined by SEM micrographs and their structure by XRD [81]. AgNPs derived from green synthesis have gained significant recognition for their potential in biomedical and pharmaceutical fields [82]. They offer the additional benefits of being environmentally friendly, cost‐efficient, easily scalable, and capable of generating higher yields than those produced through chemical means [83, 84].

### 3.3. Green ZnO NP Synthesis Based on Plants

Several writers have suggested obtaining ZnO NPs by utilizing a specific chemical composition of plant extracts as a process route [85]. According to them, instead of forming a coordinated complex, the section from plants causes the reduction of zinc (II) ions to metallic zinc [86]. The removal of the zinc precursor triggered a reaction between metallic zinc and oxygen in the solution, ultimately leading to the formation of ZnO nuclei. And they suggested that the phytoconstituents acted as a stabilizer, preventing particles from clumping together [87–90]. Ultimately, the precise method by which plant extracts facilitate the biosynthesis of ZnO NPs remains unclear, posing a significant obstacle to overcome [5]. Zinc oxide NPs were produced through an environmentally friendly approach utilizing *Agathosma betulina* plant extract as a potent chelating agent. The resulting NPs were characterized, and their critical physical properties were determined. This method represents the first successful synthesis of ZnO NPs using a fully green process [91].

### 3.4. Green AuNP Synthesis Based on Plants

The original idea behind producing AuNPs in plants was to utilize them as factories for creating such NPs [92]. Due to safety concerns, living plants are being used to make AuNPs instead of synthesizing them in a lab [93]. *Brassica junceae* developed AuNPs in its aerial portions when grown in pots with a 5 g Au/L gold chloride solution. In‐plant investigations, or phytomining, exploit the metabolic processes of living plants to produce uniformly tiny AuNPs that can be recovered using enzymes. By using this method, low‐grade soil can be converted into AuNPs via biosynthesis. One study explored using three types of alfalfa plants to in planta bioreduce Au ions to AuNPs [94, 95]. The cultivation of *Medicago sativa* and *Picea mariana* plant species to facilitate the nucleation of AuNPs has been reported [96]. The medical uses of AuNPs have been extensively investigated and are widely recognized [97]. One can produce AuNPs through chemical and physical syntheses [98]. The search for alternative large‐scale technologies that are both cost‐effective and ecologically sustainable, such as green synthesis, which uses environmentally friendly biological processes, has captured the attention of researchers worldwide [99]. The global emphasis on ecofriendly nanotechnology exploration has led to the utilization of diverse nanomaterials in suitable environmental and physical applications [100].

### 3.5. Plant‐Based Green Synthesis of Aluminum Nitrate NPs

The popularity of biosynthesis is growing due to its cost‐effective, ecofriendly, and efficient method [101]. We isolated nanopowder from *Muntingia calabura* leaf using aluminum nitrate as a precursor. Before compression molding, sisal/coir, sisal/banana, and banana/coir hybrid composites received nanopowder. Three percent nanosubstitution increased residual weight in sisal/coir, sisal/banana, and banana/coir composites, while hybrid combinations had higher degradation temperatures [102]. Using a straightforward biological reduction strategy, researchers created green‐treated aluminum oxide nanoparticles (Al_2_O_3_ NPs) and observed the influence of differing pH levels on their varying particle sizes [103]. For the first time, an ecofriendly method of producing Al_2_O_3_ NPs using extract from *Prunus yedoensis* leaves (PYLE) has been developed that can be used for nitrate removal and antibacterial purposes [104]. The Al_2_O_3_ NPs that were produced were evaluated using various standard techniques for analysis and microscopy [105].

### 3.6. Plant‐Based Green Synthesis of Iron Nanoparticles (FeNPs)

Plant substrates such as flowers, seeds, leaves, bark, and stems can be used to make bioactive components for FeNPs and zero‐valent iron nanoparticles (NZVI) [106]. The ability to scale up and be used for large‐scale production makes the plant leaf extract employed for the noble iron‐based synthesis of NPs economically advantageous [107]. Over the past decade, there has been a summary of the utilization of various plant sources, microorganisms, and ecofriendly reagents like biopolymers, hemoglobin, cellulose, and glucose for the creation of FeNPs [108]. Due to their lower energy usage, microwave and hydrothermal synthesis are considered ecofriendly methods, often called green routes [109].

### 3.7. Plant‐Based Green Synthesis of Titanium Dioxide Nanoparticles (TiO_2_ NPs)

TiO_2_ NP is among the most often used engineered NPs in consumer items based on nanotechnology [110]. To create NPs and extract titanium dioxide (TiO_2_), plants must follow an environmentally friendly process known as “green synthesis,” which makes them one of the best sources for green synthesis [111]. The *Kniphofia foliosa* plant′s root extract is used to make the TiO_2_ NPs, and because this extract provides extra electrons to TiO_2_, it is thought to have antibacterial properties [112]. The intelligent approach of using different plant extracts, fungi, and bacteria, known as the green technique, is utilized to create TiO_2_ NPs, and their role in preventing cancer is explored [113]. There has been considerable focus in the past few months on the ecofriendly production of NPs containing TiO_2_ NPs and bioreduction and capping procedures facilitated by bioactive constituents in living beings, such as bacteria and plants [114]. Several processes and techniques are used to create TiO_2_ NPs by using biogenic methods [115].

### 3.8. Mechanistic Aspects and Translation Potential

The green synthesis of NPs using plant extracts is largely governed by the presence of secondary metabolites such as polyphenols, flavonoids, terpenoids, and alkaloids. Among these, polyphenols serve as potent reducing agents that convert metal ions like Ag^+^, Au^3+^, or Cu^2+^ to their metallic NP counterparts. This redox reaction initiates the nucleation and subsequent growth of NPs. Simultaneously, these polyphenols, with their multiple hydroxyl groups, often act as capping agents by binding to NP surfaces, thereby preventing aggregation and ensuring long‐term colloidal stability [4, 116]. The structure, size, and morphology of NPs are thus directly influenced by the concentration and type of these phytochemicals present in the extract.

Proteins, polysaccharides, and other biomolecules present in plant extracts also play an essential role in capping and stabilizing synthesized NPs. These biomolecules contain functional groups such as amines, carboxylic acids, and thiols, which interact with the surface of NPs and provide a stabilizing shell. This biocapping improves biocompatibility, limits oxidation, and enhances the targeting and loading capacity of NPs for biomedical applications [6, 54]. FTIR analysis commonly confirms the presence of these functional groups, while TEM imaging shows uniformly capped particles with reduced agglomeration.

Despite strong proof‐of‐concept studies at the laboratory level, scaling up green synthesis protocols for clinical or industrial application remains a key challenge. Factors such as variability in plant phytochemical content, seasonal effects, and lack of standardized extraction methods hinder reproducibility [6]. However, some successful examples exist. For instance, AgNPs synthesized using *A. indica* and *Eclipta alba* have been employed in commercial antimicrobial sprays and coatings [20]. A few research teams have moved toward pilot‐scale production, with Good Manufacturing Practice (GMP) considerations and cytotoxicity testing in progress for pharmaceutical applications. Nevertheless, clinical translation demands stringent standardization, long‐term biosafety data, and regulatory approval processes to ensure consistency and safety [52].

In comparison to ZnO and CuO NPs, which more often fell within the 20–80 nm range, AgNPs consistently showed lower particle sizes (usually 5–30 nm) and stronger antibacterial effectiveness throughout the examined investigations. While ZnO and CuO NPs often needed greater concentrations (> 20 *μ*g/mL) to obtain equivalent effects, AgNPs manufactured utilizing polyphenol‐rich leaf extracts like *A. indica* and *E. alba* demonstrated minimum inhibitory doses as low as 3–10 *μ*g/mL. AuNPs showed modest cytotoxic efficacy but excellent biocompatibility, indicating that they are more suited for drug administration than for direct antibacterial action.

## 4. Characterization Techniques

A crucial stage in confirming the effective synthesis and biological appropriateness of NPs obtained from plants is characterization. The creation, size, shape, crystallinity, surface chemistry, and colloidal stability of NPs are all confirmed by the combined use of spectroscopic and microscopic techniques, which are complementary rather than separate analytical instruments. While diffraction‐based procedures like XRD verify crystalline structure, spectroscopic techniques like UV‐vis and FTIR mainly offer information on optical behavior and surface functional groups related to phytochemical capping. While zeta potential (*ζ*‐potential) analysis assesses surface charge and dispersion stability, microscopic methods like SEM and TEM provide direct imaging of particle shape and size distribution. Reliable characterization of NPs in practice is not dependent on a single approach but rather on correlative interpretation across numerous methods.

NP characterization is crucial in material science research. The creation of nanostructures must be verified using fundamental analytical techniques like spectroscopy and microscopy [117]. Analyses of mechanical, thermal, and densities are a few examples of characterization methods that study material qualities and structures [118]. Characterization defines materials and assesses the approach′s success. And techniques include UV‐vis, FTIR, TEM, SEM, XRD, and *ζ*‐potential/particle analysis [119]. Characterization of NPs synthesized through green methods is essential for understanding their physicochemical, morphological, and functional properties. These include size and shape (morphology), surface charge and chemistry, crystallinity, dispersion stability, and surface functionalization. Such detailed property‐based characterization enables tailoring NPs for specific biomedical applications and ensures reproducibility and regulatory viability.

### 4.1. UV‐Vis Spectroscopy

**Figure 5:**
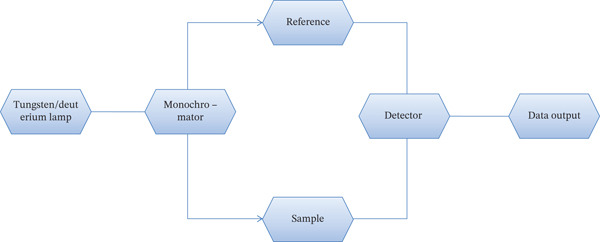
A schematic diagram of a UV‐vis spectrophotometer [122].

UV‐vis spectroscopy is the most frequently used method to assess how much extracellular green NP synthesis occurs in reacting solutions [120]. UV‐vis spectroscopy primarily provides insights into the optical properties and relative size distribution of metallic NPs through surface plasmon resonance (SPR) peaks. A shift in the SPR peak position can indicate changes in particle size, agglomeration, or surface modification. Utilizing SPR, the UV‐vis spectroscope may be used to determine the color absorption patterns of metallic NPs; in addition to assessing the concentration of NP dispersion, we also investigated the sorption, diffusion, and release properties of nanostructures [121]. A typical UV‐visible spectroscope is made up of a tungsten or deuterium lamp, a detector, and a monochromator for wavelengths in the ultraviolet and visible range [122]. A UV‐vis spectrophotometer′s schematic diagram is shown in Figure 5. To isolate particular wavelengths, the light source—a tungsten or deuterium lamp—first travels through a monochromator. After splitting into two directions, the beam passes through the sample and a reference. A detector that measures transmittance or absorbance receives both. A data output system is used to process and display the resultant data. This configuration makes it possible to precisely analyze a sample′s optical characteristics, which is very helpful when characterizing NPs.

UV‐vis spectroscopy′s underlying principle is the adsorption process [123]. The basis for the UV‐visible spectroscopy principle is the chemical compounds′ ability to absorb ultraviolet or visible light and produce unique spectra [124]. The electrons existing in materials undergo excitation when ultraviolet radiation is absorbed by them [125]. They after that oscillate between a ground state and an electrified state. It is crucial to remember that the amount of ultraviolet or visible light an electron absorbs depends on the energy difference between its ground state and its excited state. And this mechanism operates between 200 and 800 nm in the UV‐visible band. From 2 to 100 nm, different metallic NPs operate at various wavelengths. Then, 250–400 nm are needed to characterize NPs [126].

### 4.2. FTIR Spectroscopy

**Figure 6:**
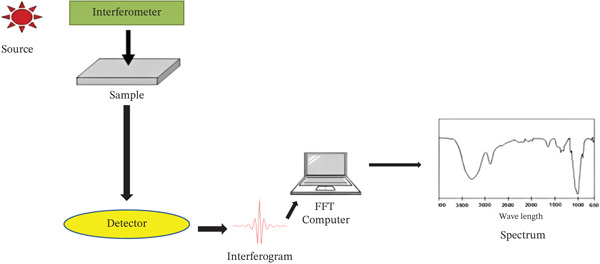
Schematic diagram of Fourier transform infrared (FTIR) spectroscopy [121].

FTIR spectroscopy is used to identify functional groups present on NP surfaces, confirming the presence of capping agents or phytochemicals responsible for stabilization. FTIR spectroscopy identifies absorption frequencies of functional groups and chemical bonds in gas, liquid, or solid samples. The spectroscope contains a source, detector, sample cell, A/D converter, amplifier, and monitor with characteristic peaks for each molecule part. Radiation passes through an interferometer to the sensor, is amplified, and is sent for Fourier transforms. Radiation turns into energy and creates a molecular spectrum, aiding in recognizing phytochemicals with FTIR [127]. Different biomolecules are discovered using specialized FTIR instruments to cap and stabilize the produced NPs [128]. A photodetector is then used to measure the optical power at the interferometer′s output as a component of the arm length difference [129]. The arm length difference is typically adjusted by carefully sliding a mirror across a certain distance, and the measured power oscillates sinusoidally. If the interferometer′s optical input were monochromatic, the optical frequency would be determined by the timing of that oscillation [130]. If the obtained light is polychromatic, the interferogram will be recorded as a superposition of various frequency components [131]. The presence of amide, hydroxyl, and carboxyl groups in FTIR spectra can indicate protein‐based capping, providing biological evidence of surface functionalization crucial for biocompatibility, as shown in Figure 6.

### 4.3. XRD

**Figure 7:**
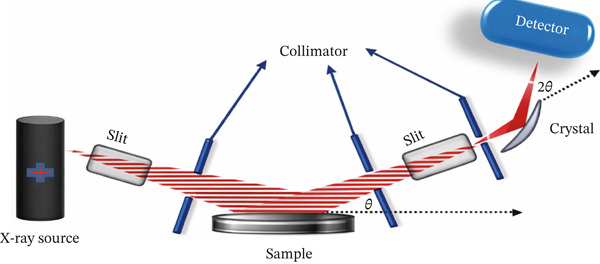
Steps of x‐ray diffraction (XRD) methods [134].

XRD is utilized to assess the crystalline structure and average crystallite size of NPs. Peaks corresponding to specific planes confirm crystalline phases such as face‐centered cubic (fcc) for AgNPs. This method allows for observing NPs′ crystal and atomic structures [132]. In a cathode ray tube, x‐rays are produced by warming the filament, which releases electrons, and accelerating those electrons toward an object by adding a voltage [133]. Monochromatic x‐ray scatter on a crystal to form a diffracted ray by interacting constructively with the atoms, as shown in Figure 7. Diffraction occurs when the scattered rays from successive planes have a path difference of multiple wavelengths [135]. The diffraction angle measurement is accomplished through Bragg′s equation [136].

2d sin θ=n λ

where *d* is the distance between the plane, *θ* is the angle of incidence, *n* is an integer, and *λ* is the beam′s wavelength. XRD data is used to calculate the NPs′ crystalline size from the Scherrer equation. For instance, AgNPs synthesized from plant extracts often exhibit crystalline domains in the range of 10–40 nm. Broader peaks may suggest smaller crystallite sizes or amorphous content. XRD is the most typical method for creating synthetic NPs and examining the crystal structure [137]. Using this characterization technique, the size of the generated crystalline NPs may also be determined [138].

### 4.4. SEM

**Figure 8:**
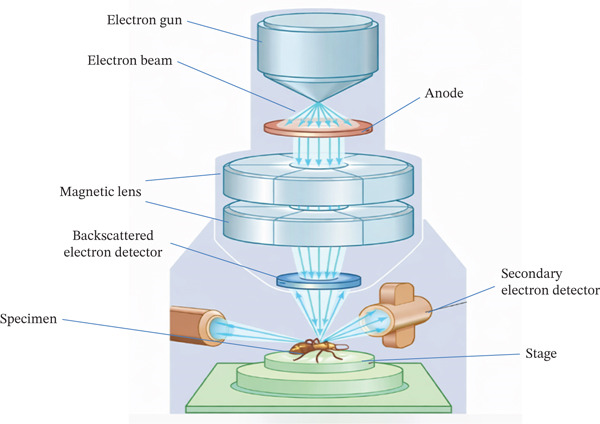
Schematic diagram of a scanning electron microscope (SEM) [144].

The optical microscope, which enlarges images with light sources (photons) and a glass lens, is comparable to this technique [139]. In contrast, an electron microscope′s incoming electron beam analyzes a material transversely to obtain information on its topography and stoichiometry [140]. During this procedure, electrons are used to form a microimage that can subsequently be examined using a magnifying glass [141]. The little column′s beat is where the electron source and focus point are placed [142]. As a result, these electrons will originate from a pillar that controls the flow of both primary and secondary electrons. To produce signals that can offer, for example, topographic data, the electronic bar interacts with the specimen. By measuring form, size, and dispersion, the morphology of the generated NPs can be observed using this technique [143]. SEM is particularly useful for assessing surface morphology, particle agglomeration, and topographical features. However, dehydration and coating processes can introduce artifacts that affect accurate shape interpretation, as highlighted in Figure 8. The virtue of synthesized NPs can, too, be seen within the magnifying lens [145].

### 4.5. TEM

**Figure 9:**
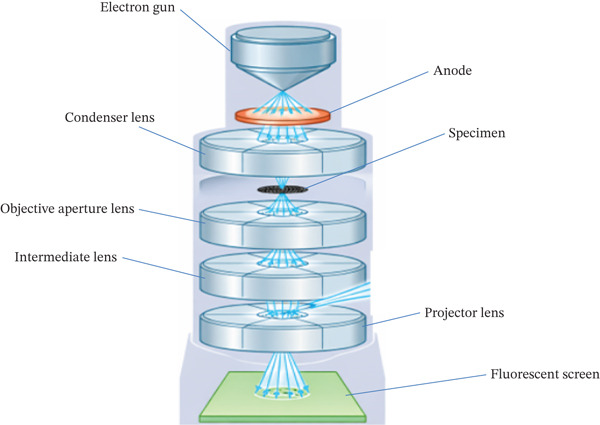
Steps of the transmission electron microscopy (TEM) method [151].

TEM microscopes, like light transmission microscopes, are primarily made to look at the interior structure of specimens [146]. TEM provides high‐resolution imaging for determining exact particle size and shape. Sizes reported via TEM for green‐synthesized silver or zinc oxide NPs typically range from 5 to 100 nm, depending on plant extract and conditions. A sample is passed through an electron beam, which creates pictures resulting from the material′s electrical interaction, in a technique called TEM [147]. The images are concentrated on the charge‐coupled device, photographic film, and fluorescent screens that can detect images [148]. TEM is the technique of choice for studying the internal microstructure of specimens, evaluating nanostructures such as particles, fibers, and thin films, and photographing atoms [149]. TEM has significant advantages for investigating nanostructures, including carbon nanotubes, graphene, and thin films [150]. While TEM offers precise size data, cross‐validation with DLS or XRD‐derived sizes is important due to differences between hydrodynamic and physical dimensions. Condenser lenses, objective aperture lenses, intermediate lenses, and projector lenses are the main components that concentrate electrons onto a fluorescent screen to create a picture in Figure 9, which depicts the steps of TEM. The procedure emphasizes the use of successive beam manipulation to accomplish microscopic high‐resolution imaging.

Characterizing a specific biomaterial is contingent upon the intricacy of its matrix, the concentration of the analyte, and its physicochemical composition [152, 153]. UV‐vis spectroscopy represents NP sizes in the 2–100 nm wavelength range of 300–800 nm. Brownish grapefruit extract confirms the Ag^+^ complex with SPR around 450–470 nm [154]. SEM photos of carbon stretches exhibit excellent morphology, but only TEM can precisely depict the form and size of NPs. Round particles between 5 and 20 nm were visible in TEM images of AgNPs [155].

Table 2 presents key techniques used to characterize NPs, organized into spectroscopy and microscopy categories. It outlines which properties—such as size distribution, shape, agglomeration state, surface area, chemical composition, and mass number—are detectable by each method. For instance, UV‐vis and DLS help analyze size distribution, while SEM and TEM reveal morphology. FTIR and XRD provide insights into surface chemistry and crystallinity. *ζ*‐Potential indicates colloidal stability. This comparative overview helps researchers choose suitable tools for comprehensive NP analysis, ensuring accurate interpretation of physical and chemical attributes essential for biomedical, environmental, and industrial applications.

**Table 2:** Overview of techniques to characterize physicochemical properties of NPs [152].

Techniques	Instruments	Mass number	Number	Size distribution	Agglomeration state	Shape	Surface area/chemical composition
Spectroscopy techniques	UV‐visible spectroscopy			✓			✓
	X‐ray diffraction			✓			✓
	FTIR spectroscopy						✓
	RAMAN spectroscopy						✓
	Atomic absorption/optical emission spectroscopy						✓
	Mass spectroscopy	✓					✓
	X‐ray photoelectron						✓
	Dynamic light scattering			✓	✓		
	Zeta potential						✓
Microscopy techniques	Scanning electron microscopy			✓		✓	
	Transmission electron microscopy			✓		✓	
	Scanning probe microscopy			✓		✓	

### 4.6. *ζ*‐Potential

*ζ*‐Potential is a key indicator of the surface charge and colloidal stability of NPs in suspension. It reflects the electrostatic potential at the slipping plane surrounding dispersed particles and is influenced by factors such as pH, ionic strength, and the presence of stabilizing agents or biomolecules on the particle surface [156]. A high absolute value of *ζ*‐potential (greater than ±30 mV) generally suggests strong repulsive forces among particles, preventing agglomeration and enhancing stability [27]. In the context of plant‐mediated green synthesis, *ζ*‐potential measurements help assess the success of NP stabilization by phytochemicals, such as flavonoids, terpenoids, and phenolic compounds. For example, AgNPs synthesized using *A. indica* leaf extract exhibited a *ζ*‐potential of −32.5 mV, indicating good colloidal stability due to capping by bioactive molecules [67]. Similarly, zinc oxide NPs synthesized from *E. alba* showed *ζ*‐potentials ranging from −22 to −38 mV, depending on pH and reaction conditions [88].

However, significant variability exists across studies due to inconsistent reporting conditions. Parameters like solvent system, temperature, buffer composition, and NP concentration affect the accuracy and comparability of *ζ*‐potential values. Therefore, standardized measurement protocols are crucial for interstudy comparisons [157]. Moreover, *ζ*‐potential also serves as an indirect indicator of surface functionalization. Shifts in *ζ*‐potential before and after conjugation with biomolecules (e.g., proteins and antibodies) can confirm surface modifications. This has implications for drug delivery applications, where surface charge influences cellular uptake, circulation time, and interaction with biological membranes [158]. *ζ*‐Potential analysis is essential for understanding NP behavior in biological and environmental systems. Despite its utility, careful consideration must be given to the interpretation of values and the influence of external factors.

Beyond physical characterization, confirming functionalization through biological assays is critical. Techniques such as FTIR and *ζ*‐potential, when paired with cytotoxicity or protein adsorption studies, validate whether plant extract–derived biomolecules remain active on the NP surface. Such confirmation is crucial for biomedical applications, including drug delivery and antimicrobial therapies.

## 5. Biomedical Applications

Numerous problems with pharmaceutical drug delivery are being solved by nanotechnology. Researchers′ interest in examining the distinctive characteristics of nanoscale materials has increased with the development of nanotechnology. In the pharmacological and medicinal industries, NPs are an appealing tool. Due to the pharmacokinetic and pharmacodynamic characteristics of numerous kinds of drugs and proteins, many researchers are searching for novel antibacterial compounds because bacteria quickly develop resistance to antibiotics [159]. In high‐demand consumer items like toothpaste, shampoo, soap, and detergents, as well as in medical and pharmaceutical applications, the noble metals gold, silver, platinum, and palladium are frequently used as NPs [160].

Because of their distinctive characteristics, AgNPs are used in a variety of commonplace human activities. In the development of sensor technology, AgNPs have been used [161]. The manufacturing of high‐performance fragile electronics, household cleaners, fabric cleaners, antireflection coatings, better heat conduction from solar energy collectors to their fuel tanks, and many more uses are among the numerous biomedical applications [162–165]. It is also more challenging to combat various virus types. The most resilient viruses and bacteria have been successfully treated using a mix of drugs and NPs. Studies conducted in 2007 by Shahverdi demonstrated that the effects of antibacterial drugs such as amoxicillin, erythromycin, clindamycin, penicillin G, and vancomycin are enhanced when nanosilver is combined with them. Nanosilver may be a key component in the treatment of AIDS patients, according to a 2015 study by Suganya et al. The scientists examined the antibacterial properties of biologically produced AgNPs against strains derived from HIV‐positive patients. Spirulina was utilized in the nanosilver synthesis. They have been changed and improved physically through the use of these particulate systems [166].

In addition to being used to make bandages, dressings, and surgical masks, nanosilver also serves as an antibacterial agent. Additionally, as an antibacterial agent, nanosilver is used in the production of bandages, dressings, and surgical masks. Due to the gradual release of silver ions, prostheses and medical equipment with a nanosilver coating guarantee long‐lasting antibacterial activity [167, 168]. CuO NPs actively fight the influenza A virus, SARS virus, and other hospital‐acquired illnesses [169].

On that scale, matter demonstrates entirely novel and unexpected qualities that obfuscate the usual distinctions between technical and scientific professions. Nanotechnology‐based solutions are frequently employed in the medical field. The most significant instances of metal NP use in pharmacology, cancer treatment, and stomatology have been described in this paper [170]. By definition, nanobiotechnology is a multistrategic method that combines nanotechnology and biotechnology to adjust the properties of therapeutic agents as the emphasis shifts from basic biological research to clinical applications. Such as the targeted administration of therapies via NPs, NPs have some peculiar properties; as a result, they can effectively diagnose and/or cure a variety of diseases, including cancer, by carefully adjusting their size, morphology, and surface properties. In addition, methods for better achieving therapeutic objectives rely on “responsive” nanomaterials that release the active components in response to specific stimuli, such as pH, redox potential, temperature, enzymes, or other variables that depend on external stimuli [171].

Green‐synthesized NPs have demonstrated broad‐spectrum utility across major biomedical applications such as antimicrobial, anticancer, wound healing, and targeted drug delivery. For example, AgNPs synthesized using *A. indica* or *E. alba* extracts have shown minimum inhibitory concentrations (MICs) ranging from 3 to 10 *μ*g/mL against *Escherichia coli* and *Staphylococcus aureus*, performing comparably or even better than their chemically synthesized counterparts [66, 171]. In anticancer evaluations, plant‐derived ZnO and AuNPs showed selective cytotoxic effects with IC_50_ values between 12 and 25 *μ*g/mL against MCF‐7 and A549 cancer cell lines, while sparing normal fibroblasts, indicating a high therapeutic index [14, 67]. In wound healing, AgNPs derived from green synthesis accelerated re‐epithelialization and collagen deposition in rodent models, demonstrating faster closure and reduced infection rates [51].

Compared to chemically synthesized NPs, green NPs generally show improved biocompatibility, biodegradability, and lower in vitro cytotoxicity due to the presence of surface‐bound phytochemicals like polyphenols and terpenoids that act as natural capping and reducing agents [20, 56]. However, some in vivo toxicity concerns have been raised at higher dosages (> 50 mg/kg), where hepatic or renal stress was noted in murine models [6]. In addition to in vitro assessments, several in vivo studies have reported favorable biosafety profiles for plant‐derived NPs. Long‐term exposure studies in rodent models have shown that green‐synthesized silver and zinc oxide NPs, administered at therapeutic doses (< 20 mg/kg), did not induce significant histopathological changes in major organs such as the liver, kidney, or spleen over observation periods of 28–90 days. These findings suggest that phytochemical capping may mitigate systemic toxicity and improve in vivo tolerability, although comprehensive chronic toxicity and biodistribution studies remain limited and warrant further investigation. Additional challenges include inconsistencies in synthesis protocols, limited scale‐up success, and a lack of phytochemical standardization, which hinder reproducibility and regulatory approval. Although no plant‐based NPs have yet reached large‐scale clinical trials, some AgNP formulations—such as gels and antimicrobial sprays—have entered preclinical and GMP‐compliant pilot production for pharmaceutical applications [10, 11].

Although green‐synthesized NPs have found utility in diverse domains such as agriculture, textiles, and environmental remediation, only those applications with direct relevance to biomedical contexts are discussed herein. Specifically, recent studies have demonstrated that metal NPs like silver (AgNPs) and zinc oxide (ZnO NPs), when integrated into medical textiles and implant coatings, provide robust antimicrobial barriers—significantly reducing risks of postoperative infections and biofilm formation on surgical implants [4, 48]. These coatings are especially crucial for orthopedic and dental implants, where microbial colonization is a leading cause of implant failure [42].

In the textile domain, biomedical relevance emerges when fabrics are modified with plant‐derived NPs to manufacture wound dressings or surgical gowns with sustained antimicrobial activity. For instance, AgNPs synthesized from *A. indica* have been embedded in cotton dressings to inhibit *S. aureus* and *E. coli* colonization, illustrating the cross‐section of textile and biomedical applications [66]. Likewise, biodegradable polymer nanocomposites integrated with ZnO NPs from *E. alba* are being explored for controlled drug‐releasing wound patches, combining flexibility with therapeutic functionality [56].

The goal of contemporary healthcare is to raise patients′ chances of survival and enhance their quality of life. To function as naturally as possible in the human body, implantable materials must be developed. Reliability and safety in usage are the essential requirements for implants comprised of artificial materials. They must not cause cancer, be toxic to living things, be inert to living tissues, be sufficiently mechanically robust, and be immune to the impacts of the internal environment of the body [172]. Implant‐associated infection is a common postoperative consequence of orthopedic surgery that commonly results in patients experiencing agony, financial difficulty, and even death [173].

### 5.1. Dental Implants and Stress Distribution

Extensive research has demonstrated that restorative materials with a high elastic modulus, such as titanium, zirconia, and certain advanced ceramics, effectively reduce stress on the abutment, dental implant, and surrounding peri‐implant bone during functional loading. These stiffer materials help distribute occlusal forces more evenly, minimizing stress concentrations that could lead to implant failure or bone resorption. Proper selection of restorative materials is essential, as it directly influences the implant′s ability to withstand excessive biting forces and parafunctional habits like bruxism. Alongside careful treatment planning and a thorough understanding of implant biomechanics, choosing materials with appropriate mechanical properties plays a crucial role in preventing complications. Since bone behavior is inversely related to load magnitude, materials that reduce excessive stress help preserve bone tissue and promote long‐term implant success. Overall, combining suitable materials with meticulous clinical protocols significantly lowers the risk of implant failure and enhances patient outcomes [174–177].

Recently, plant‐derived NPs have been explored as surface modifiers for dental implants to enhance antibacterial activity and osseointegration. Green‐synthesized silver and zirconium NPs, capped with bioactive phytochemicals, have been incorporated into titanium and zirconia implant coatings to reduce peri‐implant bacterial colonization while maintaining favorable stress distribution and mechanical integrity. Such biofunctionalized coatings offer a promising strategy to combine mechanical optimization with infection resistance in dental implant systems.

### 5.2. Metal Implants

Titanium, its alloys, specific grades of stainless steel, and other conventional metals have been used as implants in medicine. The outstanding plastic properties, great chemical stability, and biocompatibility of titanium and its alloys make them the perfect materials for making load‐bearing implants. Titanium and its alloys are also known for having high strength [178]. The insufficiently high wear and corrosion resistance, high coefficient of friction, and low bioactivity of Ti alloys, on the other hand, along with their high coefficient of friction, indicate the need for additional surface modification. Additionally, abrasion of the implant that is too strong and the entry of metal ions into the bodily environment might decrease the implant′s fixation and cause a hazardous reaction. Ti and its Ti64 alloy have been employed as biomaterials for implants since the 1950s, although their composition and surface characteristics are constantly changing. Ongoing research is being done on how thermomechanical processing affects the properties of titanium alloys. More research into developing new shape‐memory, low Young′s modulus titanium alloys [179, 180]. The danger of premature failure is higher for any material with a Young′s modulus close to that of bone. A porous metal implant would be the most effective technique to create a synthetic permanent replacement with bone′s Young′s modulus in this regard. These porous networks are adaptable to the host bone′s mechanical properties, and their porous design encourages the host bone′s fusion with them [181].

### 5.3. Ceramic Implants

An additional type of implant material is ceramic, which is produced by sintering clays and clay mixtures with mineral additions, metal oxides, and other inorganic substances. In the late 1960s, interest in using ceramics for biomedical applications first emerged as a metal‐free option with improved biocompatibility [182–186]. Bioceramic materials come in the following varieties, depending on how the body reacts to the implant: The use of bioinert ceramics in bone prosthesis is constrained by several challenges, despite the evident advantages of ceramic materials in terms of biochemical compatibility with the body when compared to metals and polymers currently employed for the restoration of the musculoskeletal system. Since it avoids the mechanical loads that lead to the resorption of the bone tissue surrounding the implant and its eventual loss, the low durability of such ceramics is a serious drawback. Recently, zirconium prosthetic implant failures have been recorded as a result of the implants′ fast crack propagation. Research is now being done on a new generation [187]. Regarding stable or bioinert ceramics, the topic of altering their surfaces to enhance the process of the implant′s osteointegration with the surrounding bone tissue is brought up. Given their inertia, giving the material antibacterial qualities to prevent adherence and multiplication of pathogenic germs is a requirement for their effective use. Modification techniques include spraying bioactive components, changing the surface topography and porosity in the area that will come into contact with bone tissue, and others. Making composite materials with new qualities from ceramics is an alternative method of altering them. Recent studies describe the use of laser ablation technology to create novel ZrO_2_ surfaces functionalized with AuNPs or Ag microparticles. Friction testing showed that neither functionalized surface integrity was impaired after implantation, and the ZrO_2_ surface was uniformly distributed with particles. This led to the new idea of creating an antibacterial surface that combines functionalization at the nano‐ and microscales. A new class of therapeutic ceramic materials with properties that are superior to those of its current analogs will be created as research in this area moves further [188].

### 5.4. Carbon Nanostructured Implants (CNIs)

The biomedical field has identified CNIs as a remarkable clinical advance because of their green synthesis production approach, alongside their remarkable biological adaptability [189]. Implants use engineering properties of carbon nanomaterials with extensive surface area, mechanical stability, and electric current capabilities to optimize integration with tissue structures [190, 191]. Plant extracts and microorganisms in green synthesis reduce manufacturing toxic waste while creating more compatible materials, which lower the body′s immune reactions [192]. The specific design of CNIs helps tissue regeneration while guiding cell adhesion at implant sites because it enables direct delivery of treatment agents, which results in better healing, together with reduced infection risks [193, 194]. The flexible nature of CNIs enables their use in orthopedic, dental, and cardiovascular implants, which require enduring stability together with perfect biological integration. Sustainable nanotechnology development of green‐synthesized CNIs creates opportunities for a medical device revolution by providing safer substitute materials that match environmental commitments and patient safety protocols [195]. Table 3 provides a comparative overview of the biological activities of green‐synthesized AgNP and AuNP. Overall, AgNPs demonstrated superior antibacterial efficacy, exhibiting lower MIC values against both Gram‐positive and Gram‐negative bacterial strains, particularly *Pseudomonas aeruginosa*.

**Table 3:** Summary of biological activities of green‐synthesized silver and gold nanoparticles [196].

Nanoparticle	Biological assay	Test model	Quantitative result	Key observation
AgNPs	Antibacterial (MIC)	MRSA, MSSA, *K. pneumoniae*	250 *μ*g/mL	Higher antibacterial potency than AuNPs
AgNPs	Antibacterial (MIC)	*P. aeruginosa*	125 *μ*g/mL	Most sensitive bacterial strain
AuNPs	Antibacterial (MIC)	MRSA, *K. pneumoniae*	1000 *μ*g/mL	Lower antibacterial efficacy
AuNPs	Antibacterial (MIC)	MSSA, *P. aeruginosa*	500 *μ*g/mL	Moderate inhibition
AgNPs	Cytotoxicity (IC_50_)	Vero cells	693.68 *μ*g/mL	Low toxicity to normal cells
AuNPs	Cytotoxicity (IC_50_)	Vero cells	661.24 *μ*g/mL	Comparable biocompatibility
AgNPs	Anticancer (IC_50_)	MCF‐7 cells	370.56 *μ*g/mL	Higher anticancer potency
AuNPs	Anticancer (IC_50_)	MCF‐7 cells	394.79 *μ*g/mL	Moderate anticancer activity
AgNPs	Antioxidant (DPPH IC_50_)	Free radical scavenging	19.7 *μ*g/mL	Strong antioxidant activity
AuNPs	Antioxidant (DPPH IC_50_)	Free radical scavenging	194.0 *μ*g/mL	Weaker antioxidant activity

In cytotoxicity assessments using Vero cells, both AgNPs and AuNPs displayed relatively high IC_50_ values, indicating low toxicity toward normal mammalian cells and favorable biocompatibility profiles. Notably, AgNPs showed enhanced anticancer activity against MCF‐7 breast cancer cells compared to AuNPs, suggesting greater therapeutic potential. In addition, AgNPs exhibited significantly stronger antioxidant activity, as reflected by lower DPPH IC_50_ values, whereas AuNPs showed comparatively weaker radical‐scavenging capacity. Collectively, these findings highlight the metal‐dependent variation in biological performance and underscore the enhanced multifunctional bioactivity of plant‐derived AgNPs relative to AuNPs.

## 6. Challenges and Future Perspectives

Despite extensive progress in green synthesis of NPs, several critical challenges continue to impede their clinical and industrial translation.

### 6.1. Regulatory and Industrialization Challenges

A major bottleneck in the commercialization of plant‐derived NPs is the lack of standardized synthesis protocols. Factors such as variations in plant phytochemical profiles, seasonal fluctuations, and inconsistent extraction techniques result in reproducibility issues [197]. These inconsistencies make it difficult to meet regulatory requirements set by agencies like the FDA or EMA, which require stringent quality control, pharmacokinetic data, and toxicological assessments [4]. Moreover, while green synthesis is environmentally friendly and cost‐effective, its industrial scalability remains limited. Challenges include sourcing consistent raw plant material, maintaining NP uniformity during scale‐up, and optimizing purification methods to meet GMP standards. Few successful examples have moved beyond the lab, and those that have remain in early pilot or preclinical stages.

### 6.2. Toxicological and Pharmacokinetic Gaps

Another significant barrier is the lack of comprehensive toxicological profiling. Although many studies report favorable in vitro biocompatibility, in vivo data on long‐term toxicity, immunogenicity, and organ‐specific accumulation are scarce [14]. Similarly, pharmacokinetic studies—critical for determining dosing, metabolism, and excretion—are limited or entirely absent in most publications. The immune response to plant‐based NPs remains poorly understood. There is an urgent need for systematic investigations into whether these NPs trigger proinflammatory reactions, cytokine release, or hypersensitivity, especially in biomedical applications like drug delivery [11].

### 6.3. Future Research Directions

To advance the clinical and industrial application of green‐synthesized NPs, future research should prioritize the standardization of synthesis protocols—including solvent type, pH, temperature, and extraction methods—to ensure interstudy reproducibility and meet regulatory standards [6]. Comprehensive pharmacokinetic and biodistribution studies in animal models are essential to predict behavior in human systems and guide therapeutic dosing [6]. Equally important is the investigation of long‐term and dose‐dependent toxicological effects under physiological conditions to ensure safety [20]. Detailed studies on immune interactions—such as cytokine profiling, complement activation, and histopathological analysis—are needed to assess potential immunogenic responses [23, 198]. To facilitate scalable production, the development of bioreactor‐based or automated synthesis platforms compliant with GMPs should be pursued [27, 46]. Additionally, creating centralized, publicly accessible databases compiling synthesis methods, biological activity, and safety data would support future meta‐analyses, improve transparency, and assist in regulatory approval processes [199]. Addressing these priorities will be crucial for translating plant‐based nanotechnology into practical, safe, and scalable solutions across medicine, agriculture, and environmental sectors.

## 7. Conclusion

The use of plant‐derived NPs offers a promising route for the creation of advanced nanomaterials with a range of uses. Numerous benefits, such as affordability, scalability, and environmental sustainability, are provided by the green synthesis techniques used in their production. Insights into the structural and functional characteristics of plant‐based NPs can be gained from the characterization methods used to evaluate their physicochemical characteristics. Applications for NPs made from plants include catalysis, energy storage, environmental remediation, healthcare, and agriculture. Due to their improved biocompatibility and capacity for targeted delivery, these NPs have shown great promise in the healthcare industry as drug delivery systems, antimicrobial agents, and anticancer agents. Plant‐based NPs have demonstrated potential in agriculture as nanofertilizers, pesticides, and growth regulators, enhancing crop sustainability and minimizing environmental impact. Additionally, their uses in environmental cleanup, catalysis, and energy storage demonstrate their adaptability and potential to address important global issues. Despite the substantial advancements made in this area, difficulties still exist. Future research must focus on standardizing synthesis procedures, improving the stability and reproducibility of NPs, and thoroughly analyzing their long‐term effects on living systems. To enable their widespread application, plant‐based NPs′ commercial viability and scalability need to be further investigated.

## Author Contributions

**Md Hosne Mobarak:** resources, writing, and visualization. **Md. Zobair Al Mahmud:** reviewing, writing, and visualization. **Amran Hossain:** editing, writing, and visualization. **Md. Arman Hossain Abir:** writing and visualization. **Nayem Hossain:** conceptualization. **Mohammad Asaduzzaman Chowdhury:** resources, supervision, visualization, and validation.

## Funding

No funding was received for this manuscript.

## Conflicts of Interest

The authors declare no conflicts of interest.

## Supporting information


**Supporting Information** Additional supporting information can be found online in the Supporting Information section.

## Data Availability

The authors confirm that the data supporting the findings of this study are available within the article.
