# Supplementary material for: Advances of Green‐Synthesized Nanoparticles for Biomedical Applications
Source: Biomed Res Int. 2026 Apr 2;2026:9207147. doi: 10.1155/bmri/9207147 (PMC13045281; doi:10.1155/bmri/9207147)
Supplement: Supplementary file 1 — Supporting Information Additional supporting information can be found online in the Supporting Information section. [file BMRI-2026-9207147-s001.docx]

**Advances of Green Synthesized Nanoparticles for Biomedical Applications**

Md Hosne Mobarak

Department of Mechanical Engineering

IUBAT-International University of Business Agriculture and Technology, Dhaka

[Email-18207023@iubat.edu](mailto:Email-18207023@iubat.edu)

Md. Zobair Al Mahmud

Department of Mechanical Engineering

IUBAT-International University of Business Agriculture and Technology, Dhaka

[Email- 20107009@iubat.edu](mailto:Email-amranhossainiubat@gmail.com)

Amran Hossain

Department of Mechanical Engineering

IUBAT-International University of Business Agriculture and Technology, Dhaka

[Email-amranhossainiubat@gmail.com](mailto:Email-amranhossainiubat@gmail.com)

Md. Arman Hossain Abir

Department of Mechanical Engineering

IUBAT-International University of Business Agriculture and Technology, Dhaka

[Email- armanavir1000@gmail.com](mailto:Email-amranhossainiubat@gmail.com)

Nayem Hossain

Department of Mechanical Engineering

IUBAT-International University of Business Agriculture and Technology, Dhaka

[Email-nayem.hossain@iubat.edu](mailto:Email-nayem.hossain@iubat.edu)

Professor Dr. Mohammad Asaduzzaman Chowdhury

Department of Mechanical Engineering

Dhaka University of Engineering and Technology (DUET), Gazipur

Email: [asadzmn2014@yahoo.com](mailto:asadzmn2014@yahoo.com)

**Abstract:**

In biomedical nanotechnology, plant-mediated production of metallic nanoparticles has become a viable and physiologically adaptable method that offers decreased toxicity, enhanced biocompatibility, and functional surface modification by phytochemical capping. In addition to altering physicochemical characteristics including size, shape, crystallinity, and surface charge, plant extracts also function as reducing, stabilizing, and capping agents, allowing for controlled nanoparticle production. Antimicrobial efficacy, cytotoxic selectivity, antioxidant activity, and interactions with mammalian cells are among the properties that significantly influence biological performance. The mechanistic knowledge of nanoparticle production and structure-activity correlations has been made easier by developments in spectroscopic, microscopic, and surface analytical methods. When taken as a whole, plant-derived nanoparticles show encouraging biomedical potential in antimicrobial therapy, anticancer applications, wound healing, and drug delivery assisted by nanocarriers. However, they also pose issues with standardization, reproducibility, and translational scalability. This semi-systematic review summarizes recent advancements in the synthesis, characterization, and biomedical applications of plant-derived nanoparticles, highlighting quantitative trends, mechanistic insights, and important knowledge gaps pertinent to future clinical translation. It covers literature from 2015 to 2024 and analyzes over 120 studies.

**Keywords:** Plants, Nanoparticles, Synthesis, Characterization, Pharmaceutical

**1. Introduction**

Since the discovery of nanomaterials, research focusing on materials with diameters of 100 nm or less has expanded rapidly across multiple scientific disciplines [1]. Nanoparticles have demonstrated different catalytic, thermal, optical, electrical, and biological capabilities used in various fields due to their high surface energy, large surface area to volume ratio, and relatively small size compared to bulk material [2]. Compared to traditional chemical and physical synthesis methods, plant-mediated green synthesis of nanoparticles is a sustainable, cost-efficient, and scale-able form that does not produce harmful side products and severe reaction conditions [3]. In this review, attention is drawn to the synthesis of nanoparticles using plant-derived biomolecules, but not microbial or enzymatic pathways.

In the current years, there has been an upsurge of the number of research directed toward sustainable nanoparticle synthesis, specifically, through the use of plants. Unlike the conventional chemical and physical methods that usually require toxic reagents and energy-dense methods in order to complete the processes, green synthesis utilizes the inherent phytochemicals in eliminating and stabilizing metallic ions at ambient conditions. The methodological path follows the green chemistry principles and favors biomedical innovation through providing biocompatible, environmentally friendly and economically accessible options [4, 5]. According to Hano and Abbasi (2022), plant-mediated synthesis has gained significant momentum due to its broad applicability and flexibility in drug delivery, tissue engineering, and antibacterial therapeutics [6]. Although this has increased, there are still inconsistencies in standardization of plant species, task preparations and synthesis procedures. This review answers these questions by evaluating the advance and deficiencies of plant-based green synthesis methods over the years 2015-2024 in critical analysis.

A crucial step in creating plant-derived nanoparticles is using the naturally occurring reducing and stabilizing compounds present in various plant components, including leaves, stems, roots, and fruits [7]. This strategy, also called "green synthesis," offers a viable and environmentally responsible replacement for traditional chemical synthesis techniques [8]. Green synthesis techniques lessen the need for dangerous chemicals and provide a scalable, affordable method for producing nanoparticles [9].

Understanding the physicochemical characteristics of plant-derived nanoparticles and confirming their suitability for particular applications require thorough characterization [10]. These nanoparticles can be characterized at the nanoscale using cutting-edge analytical techniques like spectroscopy, microscopy, and elemental analysis, which reveal their size, morphology, composition, surface properties, and stability. Such characterization enables the customization of plant-based nanoparticle properties to meet the needs of various applications [11].

Due to their extraordinary potential for drug delivery and therapeutic applications, plant-derived nanoparticles have attracted considerable interest in the pharmaceutical industry [12]. These nanoparticles have several benefits, such as controlled drug release, targeted delivery, and biocompatibility [13].Plant-derived nanoparticles have found use in dental, metal, and ceramic implants, providing unique benefits in improved biocompatibility and performance [14]. These nanoparticles can be added to improve the mechanical qualities of implant materials, such as strength and durability [15].

In spite of these benefits, green synthesis via plants, faces some challenges related to their inability to be easily repurchased over time, difficulty in increasing the manufacturing volume, and absence of standard procedures, which make it increasingly hard to gain regulatory clearance and translate into medicine. This review aims to give a thorough overview of the synthesis, characterization, and applications of plant-derived nanoparticles. We hope that by highlighting these nanoparticles' unique qualities and various uses, we can encourage more investigation into and advancements in this quickly developing area. The study of environmentally friendly and nature-inspired methods in nanotechnology shows great promise for tackling significant societal problems and opening up new horizons for cutting-edge technologies.

This semi-systematic review analyzed literature from January 2015 to February 2024 using databases such as Scopus, Web of Science, PubMed, ScienceDirect, and Google Scholar. Search terms included combinations of "green synthesis," "plant extract," "nanoparticles," "silver nanoparticles," "ZnO nanoparticles," and "biomedical applications" using Boolean operators. Articles were included if they were peer-reviewed, in English, and involved plant-mediated synthesis of nanoparticles with at least one characterization technique and biomedical relevance. Excluded were reviews, non-English texts, microbial/enzymatic synthesis studies, or those lacking experimental detail. From an initial pool of around 230 articles, 120 were selected after full-text screening. While studies often demonstrated promising results, reproducibility remains a significant issue due to inconsistent synthesis parameters—such as plant species, extract preparation methods, pH, and temperature—leading to variability in nanoparticle size, yield, and stability. For example, silver nanoparticles synthesized using Azadirachta indica frequently showed consistent outcomes, whereas other nanoparticles like ZnO and AuNPs exhibited wider variability. These findings highlight the need for standardized green synthesis protocols and detailed phytochemical profiling to enhance reproducibility and facilitate clinical translation.

2. Methodology of Review

Bionanotechnology has significantly changed contemporary science and technology [16]. The appeal of nanoparticles, particularly those made of precious metals, lies in their minute size and diverse applications in various scientific fields that serve the greater good of humanity [17]. Utilizing plants to create metal nanoparticles is advantageous as it is ecologically sound, cost-effective, and simplifies the synthesis process [18].The application of nanotechnology has emerged as a significantly promising technology [19].  Nanotechnology creates metal Nanoparticles, and biomedical technology's widespread use has gained worldwide recognition due to its versatile applications [20]. Recently, the production of metal nanoparticles by synthesis has been on the rise [21]. In-depth research has been conducted on utilising microorganisms and plants, which has been acknowledged as an intelligent and eco-friendly approach for utilizing microorganisms as functional nanofactories [22].

Using dried plant or algae extracts is a general approach for the eco-friendly production of metal nanoparticles [23].Biomolecules with unique properties that are derived from plants, algae, and microorganisms, known as natural pigments, have been utilized in the biological synthesis of NPs [24]. The research on the bioreduction of nanoparticles (NPs) using dyes has been comparatively limited [25]. Nevertheless, it has been acknowledged that pigments exhibit a robust capacity for reducing and stabilizing properties during nanoparticle synthesis through biological manufacturing[26].Currently, a wide range of plants and their byproducts are available, and several botanical insecticides have shown remarkable effectiveness and have been made available for commercial use [27]. With the advancement of science and technology, scientists are now creating herbal drugs in nano form to improve their research [28]. At this moment, an intelligent solution could be to utilize plants and their derivatives in a nano-sized form to combat the increasing population of larvae effectively [29].

**Plants Mediated Synthesis of Nanoparticles**

## **Fig. 1.**The synthesis of nanoparticles involves using various components of plants [30].

In many different sectors and areas, it is common practice to use different plant parts, such as fruits, stems, bark, seeds, latex, and callus, as illustrated in **figure 1**. The aforementioned material is used to create nanoparticles by biosynthesis [31–35].The transition of colour from lightgreen to darkbrown indicates that plants can create nanoparticles quite quickly compared to other systems [36]. A slight change in the pH and temperature of the nanoparticles can modify their shape and size [37, 38].

| 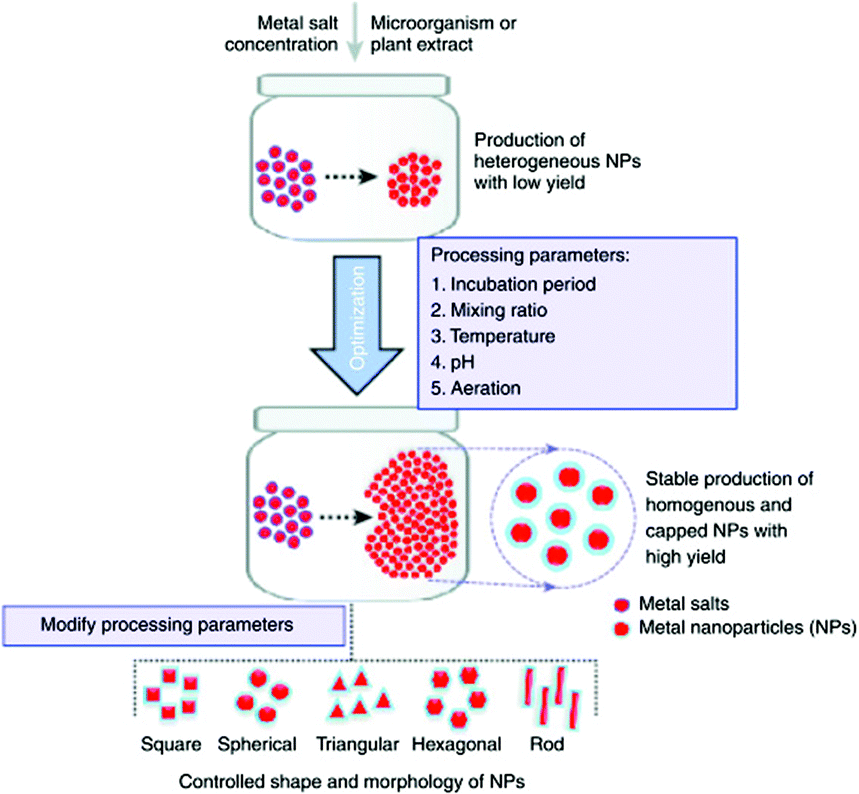 |
| --- |

**Fig.2.**The biosynthesis of metallic nanoparticles entails a series of stages [39].

**Figure 2** illustrates the optimization process in green synthesis of nanoparticles. Initially, heterogeneous nanoparticles form with low yield. By adjusting parameters like incubation time, mixing ratio, temperature, pH, and aeration, the process yields stable, homogeneous, capped nanoparticles. Shape and morphology—square, spherical, triangular, hexagonal, or rod—can be controlled by modifying processing conditions. It is well-established that diverse biological organisms produce nanoparticles with distinct physical characteristics [40]. Utilizing microbial cells' highly organized physical and biological activity is a novel technique that has recently come to light for producing metal nanoparticles [41].

**3. Plant-Based Green Synthesis**

Through various methods, including those that utilize physical and chemical strategies (known as the top-down approach), as well as biological and chemical processes (known as the bottom-up method), researchers have succeeded in synthesizing NPs [42]. Green synthesis, utilizing natural methods, has been discovered to be an environmentally responsible and less hazardous alternative to physical and chemical techniques, contributing to contamination and toxicity [43].

| 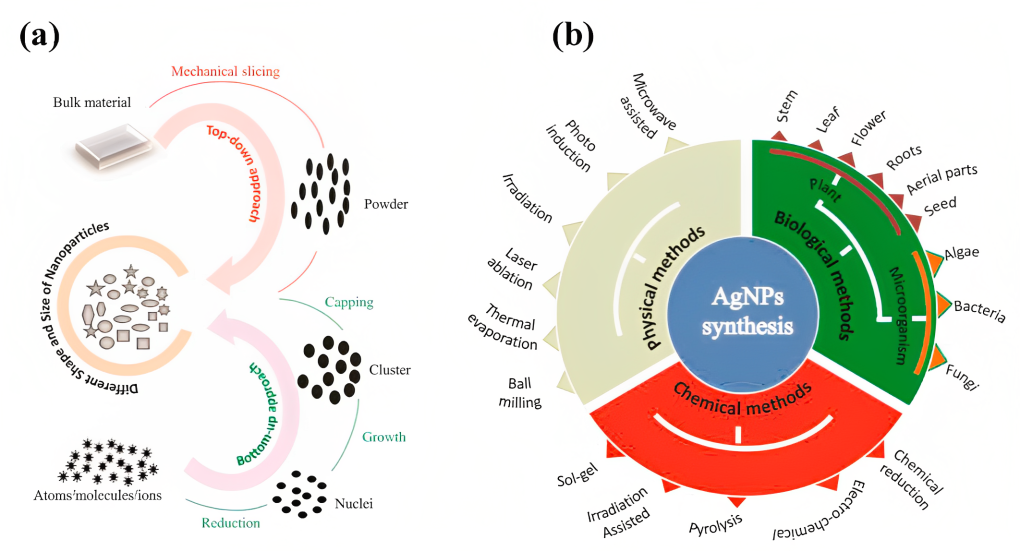 |
| --- |

## **Fig.3.** (a) Top-down and bottom-up approaches, and (b) various methods of synthesis of Nanoparticles [44].

Methods for synthesizing silver nanoparticles (AgNP) are shown in **Figure 3**. (a) demonstrates how the form and size of nanoparticles are influenced by top-down (mechanical slicing) and bottom-up (atomic reduction, growth, capping) techniques. (b) Divides synthesis methods into three categories: chemical (sol-gel, pyrolysis), biological (plants or microorganisms like bacteria, fungi, and algae), and physical (ball milling).

The production of nanomaterials can be categorized based on the approach employed, namely the bottom-up and top-down methods [45]. According to the "top-down" method, large materials or particles are gradually broken down into smaller ones using deliberate and organized physical processes like crushing, milling, grinding, lithography, and etching techniques like ion and plasma etching [46]. The primary limitations associated with this approach include elevated energy consumption levels, an incapacity to synthesize particles of a smaller size, and an imperfect surface morphology that could potentially engender significant impacts on the physical and surface features of nanomaterials [47]. The bottom-up approach entails creating nanoparticles of various sizes and shapes by arranging the atoms and molecules of a substance [48]. This technique frequently creates nanomaterials with predictable dimensions, structures, and dispersion [49]. Numerous methods, such as plant-mediated green synthesis, chemical vapour deposition (CVD), sol-gel synthesis, laser pyrolysis, microwave heating, self-assembly of monomer/polymer molecules, and supercritical hydrothermal processing, are widely used in the bottom-up approach. Following its distinct qualities, synthesis can be separated into three categories: physical, chemical, and biological processes [50].

The green synthesis methodology presents a promising approach, which entails using natural compounds to reduce, cap, and stabilise agents, thereby replacing the utilization of costly and hazardous chemical substances [51]. Numerous biological resources, including distinct plant components (roots, leaves, fruit, etc.), bacteria, fungi, and algae, among others, exist. The method above offers the possibility of environmentally friendly nanoparticle manufacturing that is biologically active [52-54]. The green production of nanoparticles utilizing botanical extracts is shown in **Figure 4**. Extract preparation, bioreduction impacted by incubation conditions, UV-Vis spectroscopy analysis of nanoparticle creation, physicochemical characterization using SEM, TEM, XRD, and FTIR, and purification and application of the generated nanoparticles are all included.

**Preparation of Botanical Extracts**

**Reduction depends on reaction mixture and incubation time**

**Nanoparticles formalization analyzed by UV- physical spectroscopy**

**Physicochemical characterization SEM ,TEM, XRD, FTIR**

**Purification and its application**

## **Fig. 4.** Steps included within the biosynthesis of nanoparticles [55].

The most effective plants for synthesising NP are those with bioaccumulation and heavy metal detoxifying abilities [56].Plant extract biomolecules have the potential to function as organic capping and reducing agents during the green synthesis of NPs. The types and portions of the plant, as well as the extraction process, affect the content of these metabolites [57].

Table 1: Plant-based Synthesis of Nanoparticles

| **Plants** | **Extracting Plant Tissues** | **Types of Nanoparticle** | **Shapes** | **Size** | **Refs** |
| --- | --- | --- | --- | --- | --- |
| Phorbia Prostrata | Leaves | Silver and Titanium dioxide (TiO2) | Spherical | Silver 10–15 (nm); TiO2, 81.7–84.7 (nm) | [58] |
| Sargassum | Alga | Palladium | Octahedral | 5 –10 (nm) | [59] |
| Ginkgo biloba | Leaves | Copper | Spherical | 15–20 (nm) | [60] |
| Panax ginseng | Root | Silver and gold | Spherical | Silver, 10–30(nm); gold, 10–40(nm) | [61] |
| Red ginseng | Root | Silver | Spherical | 10–30 (nm) | [62] |
| Cocos nucifera | Leaves | Lead | Spherical | 47 (nm) | [63] |
| Banana | Peel | Cadmium sulfide | – | 1.48 (nm) | [64] |
| Citrus medica | Fruit | Copper | – | 20 (nm) | [65] |
| Orange and pineapple | Fruits | Silver | Spherical | 10–300 (nm) | [66] |
| Gardenia jasminoides | Leaves | Iron | Rock like appearance | 32 (nm) | [67] |
| Azadirachta indica | Leaves | Silver | - | 41–60 (nm) | [68] |
| Nigella sativa | Leaves | Silver | Spherical | 15 (nm) | [69] |
| Catharanthus roseus | Leaves | Palladium | Spherical | 40 (nm) | [70] |
| Pistacia atlantica | Seeds | Silver | Spherical | 27 (nm) | [71] |
| Nyctanthes arbortristis | Flower | Silver | – | – | [72] |
| Artocarpus gomezianus | Fruit | Zinc | Spherical | > 20 (nm) | [73] |
| Cymbopogon citratus | Leaves | Gold | triangular, hexagonal, spherical, and rod | 20–50 (nm) | [74] |

From **Table 1**, it is evident that some plant extracts are more effective than others in producing smaller or more stable nanoparticles. For instance, banana peel extract yielded cadmium sulfide nanoparticles as small as 1.48 nm, while Sargassum alga produced palladium nanoparticles averaging 5–10 nm. In contrast, Cocos nucifera leaf extract resulted in relatively larger lead nanoparticles around 47 nm. These differences are likely due to the varying phytochemical compositions and reducing capacities of the plant extracts, which influence nucleation and growth mechanisms during synthesis. Such comparative data help in selecting optimal plant sources for desired nanoparticle characteristics.

# 3.1 Copper and Copper Oxide Nanoparticle Synthesis through Plant-Based Green Method

Plants are rich in phytochemicals and secondary metabolites that can be utilized as valuable bioresources to synthesise Cu and CuO NPs [75, 76]. Phenols and flavonoids are the primary phytochemicals present in various parts of plants like leaves, roots, shoots, stems, flowers, and fruits [77]. These phenols contain hydroxyl and ketone groups which aid with iron chelation and ultimately exhibit potent antioxidant characteristics, according to [78]. Using this environmentally friendly approach, the stability of NPs was enhanced while also preventing their aggregation and distortion, creating an opportunity for the absorption of phytochemicals onto the NPs' surface. This absorption process increased the NPs' reaction rate [79]. Utilizing green synthesis techniques, as opposed to conventional chemical and physical techniques, produces copper oxide nanoparticles, commonly known as CuO NPs, in a way that is significantly more ecologically conscious and safe [80]. Researchers are currently studying the impact of copper oxide nanoparticles, produced through environmentally friendly means, on the green peach Aphid [81].

# 3.2 Green Silver Nanoparticle Synthesis Based on Plants

Extracts of A. millefolium were used to reduce and stabilize SNPs [82]. The plant was gathered at Rawalakot and given to the university's herbarium there. Before being turned into powder, the plant was first washed and dried. To create plant extracts, 250mL of distilled water, ethanol, and methanol were added to 25g of powder and vigorously shaken for 24 hours at room temperature. After filtering with Whatman number 1 filter paper and filtrate were used to reduce silver ions in the AgNO_3_ solution. The creation of SNPs was shown by the brown hue that resulted from the reaction of 20 mL of plant extract with 80 mL of AgNO3 (1 mM) solution over 24 hours at room temperature. The morphology of SNPs was examined by SEM micrographs and their structure by X-ray diffraction [83]. AgNPs derived from green synthesis have gained significant recognition for their potential in biomedical and pharmaceutical fields [84]. They offer the additional benefits of being environmentally friendly, cost-efficient, easily scalable, and capable of generating higher yields than those produced through chemical means [85, 86].

**3.3 *Green ZnO nanoparticle synthesis based on plants***

Several writers have suggested obtaining ZnO NPs by utilizing a specific chemical composition of plant extracts as a process route [87]. According to them, instead of forming a coordinated complex, the section from plants causes the reduction of zinc (II) ions to metallic zinc [88]. The removal of the zinc precursor triggered a reaction between metallic zinc and oxygen in the solution, ultimately leading to the formation of ZnO nuclei. And they suggested that the phyto-constituents acted as a stabilizer, preventing particles from clumping together [89-92]. Ultimately, the precise method by which plant extracts facilitate the biosynthesis of ZnO nanoparticles remains unclear, posing a significant obstacle to overcome [93]. Zinc oxide nanoparticles were produced through an environmentally friendly approach utilizing Agathosma betulina plant extract as a potent chelating agent. The resulting nanoparticles were characterized, and their critical physical properties were determined. This method represents the first successful synthesis of ZnO nanoparticles using a fully green process [94].

# 3.4 Green gold nanoparticle synthesis based on plants

The original idea behind producing AuNPs in plants was to utilize them as factories for creating such Nanoparticles [95]. Due to safety concerns, living plants are being used to make gold nanoparticles instead of synthesizing them in a lab [96]. *Brassica junceae* developed AuNPs in its aerial portions when grown in pots with a 5 g Au/L gold chloride solution. In-plant investigations, or phytomining, exploit the metabolic processes of living plants to produce uniformly tiny AuNPs that can be recovered using enzymes. By using this method, low-grade soil can be converted into AuNPs via biosynthesis. One study explored using three types of Alfalfa plants to in planta bioreduce Au ions to AuNPs [97, 98]. The cultivation of Medicago sativa and Picea mariana plant species to facilitate the nucleation of AuNPs has been reported [99]. The medical uses of gold nanoparticles (AuNPs) have been extensively investigated and are widely recognized [100]. One can produce AuNPs through chemical and physical synthesis [101]. The search for alternative large-scale technologies that are both cost-effective and ecologically sustainable, such as green synthesis which uses environmentally friendly biological processes, has captured the attention of researchers worldwide [102]. The global emphasis on eco-friendly nanotechnology exploration has led to the utilization of diverse nanomaterials in suitable environmental and physical applications [103].

# 3.5 Plant-Based Green Synthesis of Aluminum Nitrate Nanoparticles

The popularity of biosynthesis is growing due to its cost-effective, eco-friendly, and efficient method [104]. We isolated nanopowder from *'Muntingia calabura'* leaf using aluminium nitrate as a precursor. Before compression moulding, sisal/coir, sisal/banana, and banana/coir hybrid composites received nanopowder. 3% nano substitution increased residual weight in sisal/coir, sisal/banana, and banana/coir composites, while hybrid combinations had higher degradation temperatures [105].Using a straightforward biological reduction strategy, researchers created green-treated aluminium oxide nanoparticles (Al2O3 NPs) and observed the influence of differing pH levels on their varying particle sizes [106]. For the first time, an eco-friendly method of producing Al2O3 nanoparticles using extract from Prunus yedoensis leaves (PYLE) has been developed that can be used for nitrate removal and antibacterial purposes [107]. The Al2O3 NPs that were produced were evaluated using various standard techniques for analysis and microscopy [108].

# 3.6 Plant-Based Green Synthesis of Iron Nanoparticles

Plant substrates such as flowers, seeds, leaves, barks, and stems can be used to make bioactive components for iron nanoparticles (FeNPs) and zero-valent iron nanoparticles (NZVI) [109]. The ability to scale up and be used for large-scale production makes the plant leaf extract employed for the noble iron-based synthesis of nanoparticles economically advantageous [110]. Over the past decade, there has been a summary of the utilization of various plant sources, microorganisms, and eco-friendly reagents like biopolymers, haemoglobin, cellulose, and glucose for the creation of iron Nanoparticles [111]. Due to their lower energy usage, microwave and hydrothermal synthesis are considered eco-friendly methods, often called green routes [112].

# 3.7 Plant-based green synthesis of titanium dioxide nanoparticles

TiO_2_NP is among the most often used engineered nanoparticles in consumer items based on nanotechnology [113]. To create nanoparticles (NPs) and extract titanium dioxide (TiO_2_), plants must follow an environmentally friendly process known as "green synthesis," which makes them one of the best sources for green synthesis [114]. The *Kniphofia foliosa* plant's root extract is used to make the TiO_2_NPs, and because this extract provides extra electrons to TiO_2_, it is thought to have antibacterial properties [115]. The intelligent approach of using different plant extracts, fungi, and bacteria, known as the green technique, is utilized to create Titanium nanoparticles (TiO_2_-NP’s), and their role in preventing cancer is explored [116]. There has been considerable focus in the past few months on the eco-friendly production of nanoparticles containing titanium dioxide (TiO_2_ NPs) and bio-reduction and capping procedures facilitated bybioactive constituents in living beings such as bacteria and plants [117]. The several processes and techniques used to create TiO_2_ nanoparticles by using biogenic methods [118].

### **3.8 Mechanistic Aspects and Translation Potential**

The green synthesis of nanoparticles using plant extracts is largely governed by the presence of secondary metabolites such as **polyphenols, flavonoids, terpenoids,** and **alkaloids.** Among these, polyphenols serve as potent reducing agents that convert metal ions like Ag⁺, Au³⁺, or Cu²⁺ to their metallic nanoparticle counterparts. This redox reaction initiates the nucleation and subsequent growth of nanoparticles. Simultaneously, these polyphenols, with their multiple hydroxyl groups, often act as **capping agents** by binding to nanoparticle surfaces, thereby preventing aggregation and ensuring long-term colloidal stability [119, 120]. The structure, size, and morphology of nanoparticles are thus directly influenced by the concentration and type of these phytochemicals present in the extract.

Proteins, polysaccharides, and other biomolecules present in plant extracts also play an essential role in **capping and stabilizing** synthesized nanoparticles. These biomolecules contain functional groups such as amines, carboxylic acids, and thiols, which interact with the surface of nanoparticles and provide a stabilizing shell. This bio-capping improves biocompatibility, limits oxidation, and enhances the targeting and loading capacity of nanoparticles for biomedical applications [121, 122]. FTIR analysis commonly confirms the presence of these functional groups, while TEM imaging shows uniformly capped particles with reduced agglomeration.

Despite strong proof-of-concept studies at the laboratory level, **scaling up green synthesis protocols** for clinical or industrial application remains a key challenge. Factors such as variability in plant phytochemical content, seasonal effects, and lack of standardized extraction methods hinder reproducibility [123]. However, some successful examples exist. For instance, silver nanoparticles synthesized using Azadirachta indica and Eclipta alba have been employed in **commercial antimicrobial sprays and coatings [124]**. A few research teams have moved toward **pilot-scale production**, with GMP considerations and cytotoxicity testing in progress for pharmaceutical applications. Nevertheless, clinical translation demands stringent standardization, long-term biosafety data, and regulatory approval processes to ensure consistency and safety [125].

In comparison to ZnO and CuO nanoparticles, which more often fell within the 20–80 nm range, silver nanoparticles consistently showed lower particle sizes (usually 5–30 nm) and stronger antibacterial effectiveness throughout the examined investigations. While ZnO and CuO nanoparticles often needed greater concentrations (>20 µg/mL) to obtain equivalent effects, AgNPs manufactured utilizing polyphenol-rich leaf extracts like Azadirachta indica and *Eclipta alba* demonstrated minimum inhibitory doses as low as 3–10 µg/mL. Gold nanoparticles showed modest cytotoxic efficacy but excellent biocompatibility, indicating that they are more suited for drug administration than for direct antibacterial action.

**4. Characterization Techniques**

A crucial stage in confirming the effective synthesis and biological appropriateness of nanoparticles obtained from plants is characterization. The creation, size, shape, crystallinity, surface chemistry, and colloidal stability of nanoparticles are all confirmed by the combined use of spectroscopic and microscopic techniques, which are complimentary rather than separate analytical instruments. While diffraction-based procedures like XRD verify crystalline structure, spectroscopic techniques like UV-Vis and FTIR mainly offer information on optical behavior and surface functional groups related to phytochemical capping. While zeta potential analysis assesses surface charge and dispersion stability, microscopic methods like SEM and TEM provide direct imaging of particle shape and size distribution. Reliable characterization of nanoparticles in practice is not dependent on a single approach, but rather on correlative interpretation across numerous methods.

NPs characterization is crucial in material science research. The creation of nanostructures must be verified using fundamental analytical techniques like spectroscopy and microscopy [126]. Analyses of mechanical, thermal and densities are a few examples of characterization methods that study material qualities and structures [127]. Characterization defines materials and assesses approach success. And techniques include UV-vis, FTIR, TEM, SEM, XRD, and zeta potential/particle analysis [128]. Characterization of nanoparticles synthesized through green methods is essential for understanding their physicochemical, morphological, and functional properties. These include size and shape (morphology), surface charge and chemistry, crystallinity, dispersion stability, and surface functionalization. Such detailed property-based characterization enables tailoring nanoparticles for specific biomedical applications and ensures reproducibility and regulatory viability.

# 4.1 UV–vis spectroscopy

UV-vis spectroscopy is the most frequently used method to assess how much extracellular green NP synthesis occurs in reacting solutions [129]. UV–Vis spectroscopy primarily provides insights into the optical properties and relative size distribution of metallic nanoparticles through surface plasmon resonance (SPR) peaks. A shift in SPR peak position can indicate changes in particle size, agglomeration, or surface modification. Utilizing surface Plasmon resonance, the UV-vis spectroscope may be used to determine the colour absorption patterns of metallic nanoparticles (NPs); in addition to assessing the concentration of NP dispersion, we also investigated the sorption, diffusion, and release properties of nanostructures [130]. A typical UV-visible spectroscope is made up of a tungsten or deuterium lamp, a detector, and a monochromator for wavelengths in the ultraviolet and visible range [131]. A UV-vis spectrophotometer's schematic diagram is shown in **Figure 5**. To isolate particular wavelengths, the light source—a tungsten or deuterium lamp—first travels through a monochromator. After splitting into two directions, the beam passes through the sample and a reference. A detector that measures transmittance or absorbance receives both. A data output system is used to process and display the resultant data. This configuration makes it possible to precisely analyze a sample's optical characteristics, which is very helpful when characterizing nanoparticles.

| **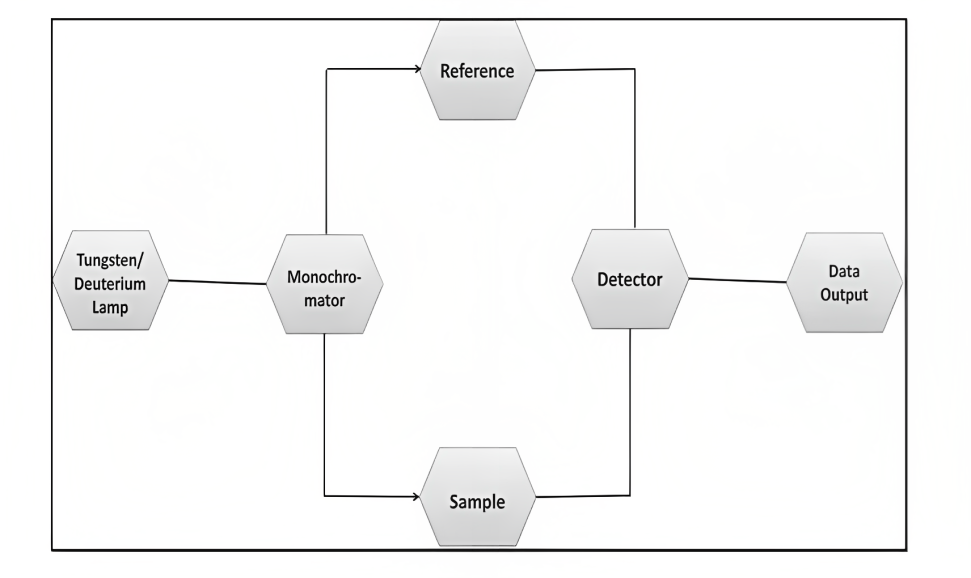** |
| --- |

**Fig. 5.** A schematic diagram of UV–vis spectrophotometer [131].

UV-vis spectroscopy's underlying principle is the adsorption process [132]. The basis for the UV-visible spectroscopy principle is the chemical compounds' ability to absorb ultraviolet or visible light and produce unique spectra [133]. The electrons existing in materials undergo excitation when ultraviolet radiation is absorbed by it [134]. They after that oscillate between a ground state and an electrified state. It is crucial to remember that the amount of ultraviolet or visible light an electron absorbs depends on the energy difference between its ground state and its excited state. And this mechanism operates between 200 and 800 nm in the UV-visible band. From 2 to 100 nm, different metallic nanoparticles operate at various wavelengths. 250–400 nanometers are needed to characterize Nanoparticles [135].

# 4.2. Fourier transforms infrared (FTIR) spectroscopy

FTIR spectroscopy is used to identify functional groups present on nanoparticle surfaces, confirming the presence of capping agents or phytochemicals responsible for stabilization. FT-IR spectroscopy identifies absorption frequencies of functional groups and chemical bonds in gas, liquid, or solid samples. The spectroscope contains a source, detector, sample cell, A/D converter, amplifier, and monitor with characteristic peaks for each molecule part. Radiation passes through an interferometer to the sensor, is amplified, and sent for Fourier transforms. Radiation turns into energy and creates a molecular spectrum, aiding in recognizing phytochemicals with FTIR [136]. Different biomolecules are discovered using specialized FTIR instruments to cap and stabilize the produced Nanoparticles [137]. A photodetector is then used to measure the optical power at the interferometer's output as a component of the arm length difference [138]. The arm length difference is typically adjusted by carefully sliding a mirror across a certain distance, and the measured power oscillates sinusoidally.If the interferometer's optical input were monochromatic, the optical frequency would be determined by the timing of that oscillation. [139]. If the obtained light is polychromatic, the interferogram will be recorded as a superposition of various frequency components [140]. The presence of amide, hydroxyl, and carboxyl groups in FTIR spectra can indicate protein-based capping, providing biological evidence of surface functionalization crucial for biocompatibility as shown in **Figure 6.**


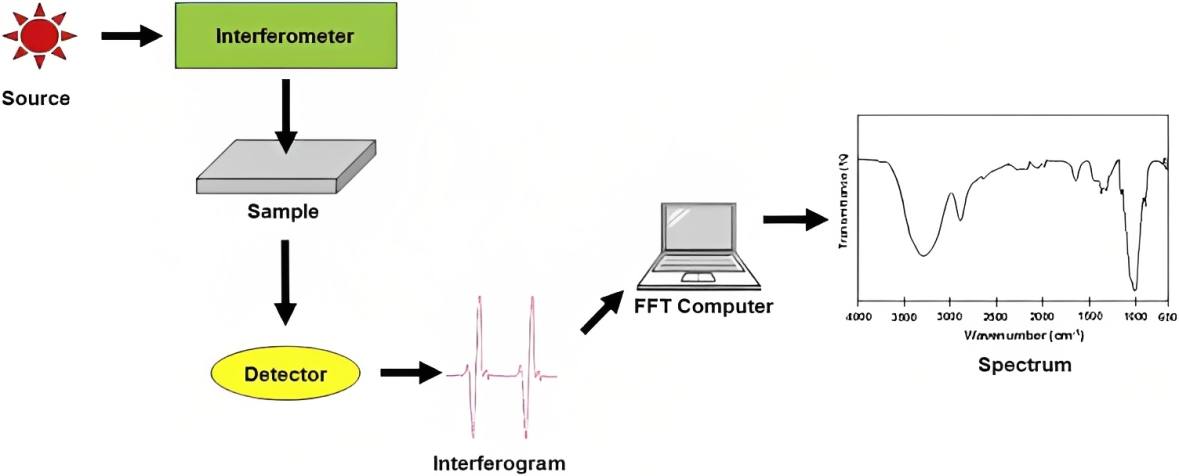


**Fig. 6.** Schematic Diagram of Fourier transform infrared (FTIR) spectroscopy [130].

# 4.3 X-ray diffraction (XRD)

XRD is utilized to assess the crystalline structure and average crystallite size of nanoparticles. Peaks corresponding to specific planes confirm crystalline phases such as face-centered cubic (fcc) for silver nanoparticles. This method allows for observing nanoparticles' crystal and atomic structures [142]. In a cathode ray tube, X-rays are produced by warming the filament, which releases electrons, and accelerating those electrons toward an object by adding a voltage [143]. Monochromatic X-rays scatter on a crystal to form a diffracted ray by interacting constructively with the atoms, as shown in **Figure 7.** Diffraction occurs when the scattered rays from successive planes have a path difference of multiple wavelengths [144]. The diffraction angle measurement is accomplished through Bragg's equation [145].

2d sin θ = n λ

Where d is the distance between the plane, is the angle of incidence, n is an integer, and λ is the beam's wavelength. XRD data is used to calculate the NPs' crystalline size from the Scherer equation. For instance, silver nanoparticles synthesized from plant extracts often exhibit crystalline domains in the range of 10–40 nm. Broader peaks may suggest smaller crystallite sizes or amorphous content. X-ray diffraction is the most typical method for creating synthetic nanoparticles and examining the crystal structure [1146]. Using this characterization technique, the size of the generated crystalline nanoparticles may also be determined [147].


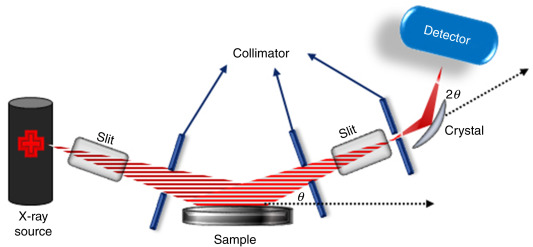


## **Fig. 7.** Steps of X-ray Diffraction (XRD) methods [1148].

# 4.4 Scanning Electron Microscope (SEM)

The optical microscope, which enlarges images with light sources (photons) and a glass lens, is comparable to this technique [149]. In contrast, an electron microscopes incoming electron beam analyzes a material transversely to obtain information on its topography and Stoichiometry [150]. During this procedure, electrons are used to form a micro-image that can subsequently be examined using a magnifying glass [151]. The little column's beat is where the electron source and focus point are placed [152]. As a result, these electrons will originate from a pillar that controls the flow of both primary and secondary electrons. To produce signals that can offer,an example, topographic data, the electronic bar interacts with the specimen. By measuring form, size, and dispersion, The morphology of the generated nanoparticles can be observed using this technique [153]. SEM is particularly useful for assessing surface morphology, particle agglomeration, and topographical features. However, dehydration and coating processes can introduce artifacts that affect accurate shape interpretation as highlighted in **Figure 8**. The virtue of synthesized nanoparticles can, too, be watched within the magnifying lens [154].


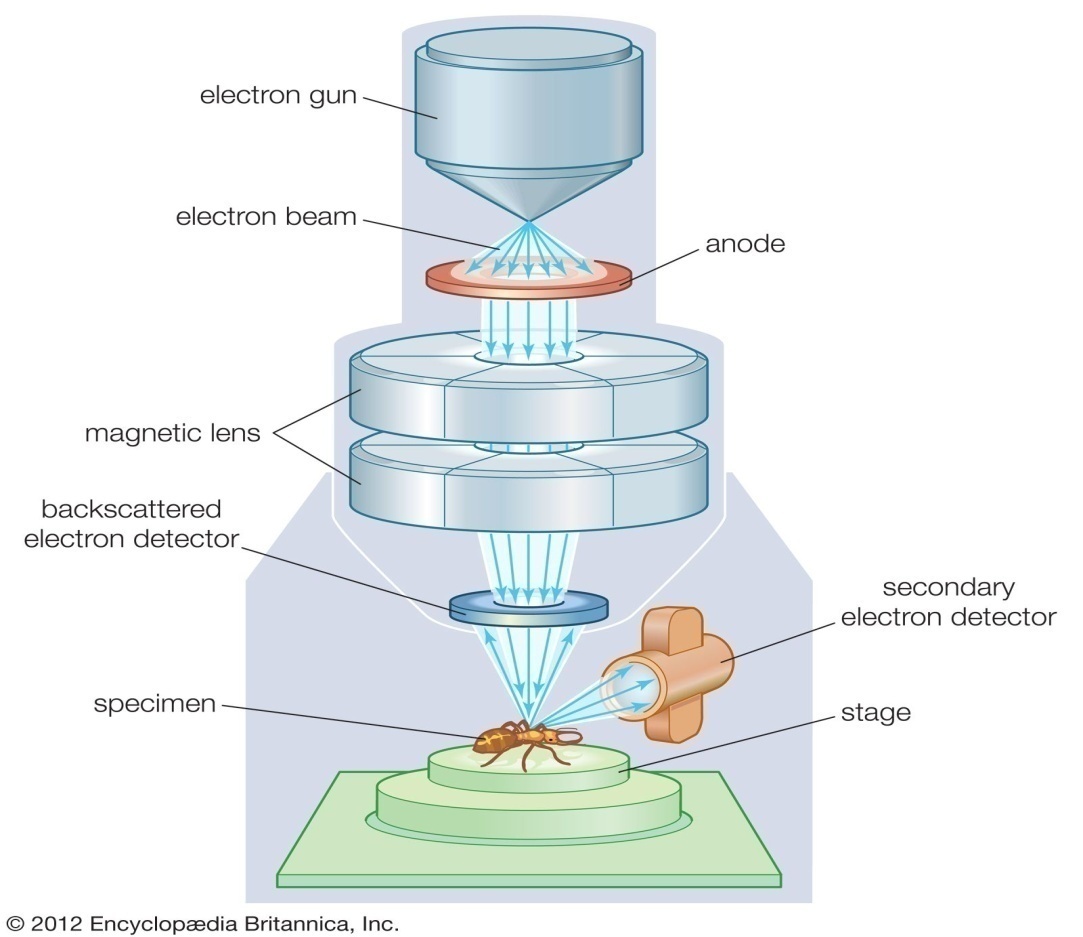


## **Fig. 8.** Schematic Diagram of Scanning Electron Microscope(SEM)[155].

# 4.5 Transmission electron microscopy (TEM)

TEM microscopes, like light transmission microscopes, are primarily made to look at the interior structure of specimens [156]. TEM provides high-resolution imaging for determining exact particle size and shape. Sizes reported via TEM for green-synthesized silver or zinc oxide nanoparticles typically range from 5 to 100 nm, depending on plant extract and conditions. A sample is passed through an electron beam, which creates pictures resulting from the material's electrical interaction, in a technique called transmission electron microscopy (TEM) [157]. The images are concentrated on the charge-coupled device, photographic film, and fluorescent screens that can detect images [158]. Transmission electron microscopy (TEM) is the technique of choice for studying the internal microstructure of specimens, evaluating nanostructures such as particles, fibres, and thin films, and photographing atoms [159]. TEM has significant advantages for investigating nanostructures, including carbon nanotubes, graphene, and thin films [160]. While TEM offers precise size data, cross-validation with DLS or XRD-derived sizes is important due to differences between hydrodynamic and physical dimensions. Condenser lenses, objective aperture lenses, intermediate lenses, and projector lenses are the main components that concentrate electrons onto a fluorescent screen to create a picture in **Figure 9**, which depicts the steps of Transmission Electron Microscopy (TEM). The procedure emphasizes the use of successive beam manipulation to accomplish microscopic high-resolution imaging.

| 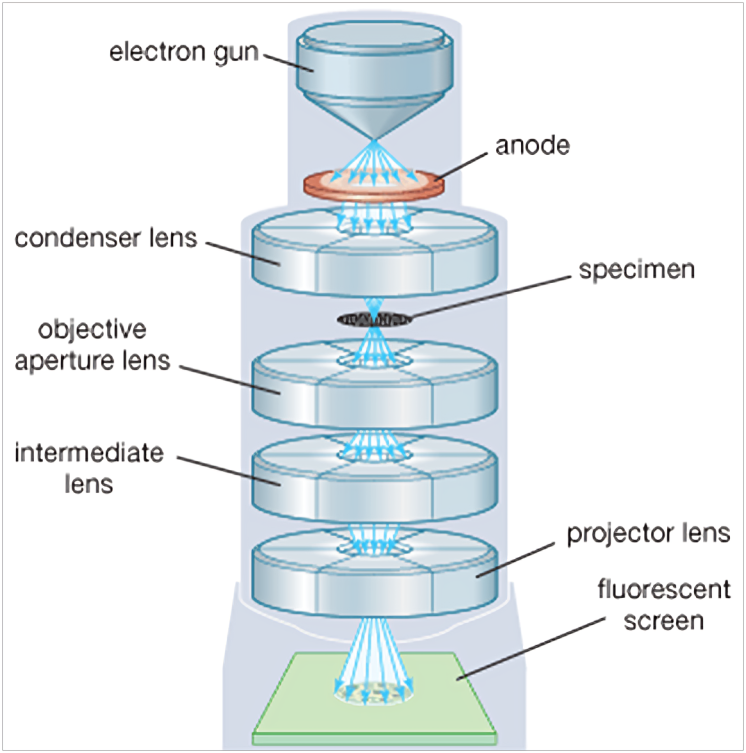 |
| --- |

## **Fig. 9.** Steps of Transmission Electron Microscopy (TEM) method [161].

Characterizing a specific biomaterial is contingent upon the intricacy of its matrix, the concentration of the analytic, and its physio-chemical composition [162, 163]. UV-vis spectroscopy represents NPs sizes 2-100 nm wavelength range 300-800 nm. Brownish grapefruit extract confirms the Ag^+^ complex with surface Plasmon resonance around 450-470 nm [164]. SEM photos of carbon stretches exhibit excellent morphology, but only TEM can precisely depict the form and size of NPs. Round particles between 5 and 20 nm were visible in TEM images of Ag NPs [165].

**Table 2:** Overview of techniques to characterize physicochemical properties of NPs [162].

| Techniques | Instruments | Mass Number | Number | Size Distribution | Agglomeration State | Shape | Surface Area / Chemical Composition |
| --- | --- | --- | --- | --- | --- | --- | --- |
| Spectroscopy techniques | UV visible Spectroscopy |  |  | ✓ |  |  | ✓ |
|  | X-ray Diffraction |  |  | ✓ |  |  | ✓ |
|  | FT-IR spectroscopy |  |  |  |  |  | ✓ |
|  | RAMAN spectroscopy |  |  |  |  |  | ✓ |
|  | Atomic absorption/optical emission spectroscopy |  |  |  |  |  | ✓ |
|  | Mass spectroscopy | ✓ |  |  |  |  | ✓ |
|  | X-ray photoelectron |  |  |  |  |  | ✓ |
|  | Dynamic light Scattering |  |  | ✓ | ✓ |  |  |
|  | Zeta potential |  |  |  |  |  | ✓ |
| Microscopy techniques | Scanning electron Microscopy |  |  | ✓ |  | ✓ |  |
|  | Transmission electron microscopy |  |  | ✓ |  | ✓ |  |
|  | Scanning probe microscopy |  |  | ✓ |  | ✓ |  |

**Table 2** presents key techniques used to characterize nanoparticles, organized into spectroscopy and microscopy categories. It outlines which properties—such as size distribution, shape, agglomeration state, surface area, chemical composition, and mass number—are detectable by each method. For instance, UV-Vis and DLS help analyze size distribution, while SEM and TEM reveal morphology. FTIR and XRD provide insights into surface chemistry and crystallinity. Zeta potential indicates colloidal stability. This comparative overview helps researchers choose suitable tools for comprehensive nanoparticle analysis, ensuring accurate interpretation of physical and chemical attributes essential for biomedical, environmental, and industrial applications.

### **4.6 Zeta Potential**

Zeta potential (ζ-potential) is a key indicator of the surface charge and colloidal stability of nanoparticles in suspension. It reflects the electrostatic potential at the slipping plane surrounding dispersed particles and is influenced by factors such as pH, ionic strength, and the presence of stabilizing agents or biomolecules on the particle surface [166]. A high absolute value of zeta potential (greater than ±30 mV) generally suggests strong repulsive forces among particles, preventing agglomeration and enhancing stability [167]. In the context of plant-mediated green synthesis, zeta potential measurements help assess the success of nanoparticle stabilization by phytochemicals, such as flavonoids, terpenoids, and phenolic compounds. For example, silver nanoparticles synthesized using Azadirachta indica leaf extract exhibited a zeta potential of –32.5 mV, indicating good colloidal stability due to capping by bioactive molecules [168]. Similarly, zinc oxide nanoparticles synthesized from Eclipta alba showed zeta potentials ranging from –22 to –38 mV, depending on pH and reaction conditions [169].

However, significant variability exists across studies due to inconsistent reporting conditions. Parameters like solvent system, temperature, buffer composition, and nanoparticle concentration affect the accuracy and comparability of zeta potential values. Therefore, standardized measurement protocols are crucial for inter-study comparisons [170]. Moreover, zeta potential also serves as an indirect indicator of surface functionalization. Shifts in zeta potential before and after conjugation with biomolecules (e.g., proteins, antibodies) can confirm surface modifications. This has implications for drug delivery applications, where surface charge influences cellular uptake, circulation time, and interaction with biological membranes [171]. Zeta potential analysis is essential for understanding nanoparticle behavior in biological and environmental systems. Despite its utility, careful consideration must be given to the interpretation of values and the influence of external factors.

Beyond physical characterization, confirming functionalization through biological assays is critical. Techniques such as FTIR and zeta potential, when paired with cytotoxicity or protein adsorption studies, validate whether plant-extract-derived biomolecules remain active on the nanoparticle surface. Such confirmation is crucial for biomedical applications, including drug delivery and antimicrobial therapies.

**5. Biomedical Applications**

Numerous problems with pharmaceutical drug delivery are being solved by nanotechnology. Researchers' interest in examining the distinctive characteristics of nanoscale materials has increased with the development of nanotechnology. In the pharmacological and medicinal industries, nanoparticles are an appealing tool. Due to the pharmacokinetic and pharmacodynamic characteristics of numerous kinds of drugs and proteins, many researchers are searching for novel antibacterial compounds because bacteria quickly develop resistance to antibiotics [172]. In high-demand consumer items like toothpaste, shampoo, soap, and detergents as well as in medical and pharmaceutical applications, the noble metals gold, silver, platinum, and palladium are frequently used as nanoparticles [173].

Because of their distinctive characteristics, silver nanoparticles are used in a variety of commonplace human activities. In the development of sensor technology, silver nanoparticles have been used [174], The manufacturing of high-performance fragile electronics, household cleaners, fabric cleaners, antireflection coatings, better heat conduction from solar energy collectors to their fuel tanks, and many more uses are among the numerous biomedical applications [175–178]. It is also more challenging to combat various virus types. The most resilient viruses and bacteria have been successfully treated using a mix of drugs and nanoparticles. Studies conducted in 2007 by Shahverdi demonstrated that the effects of antibacterial drugs such as amoxicillin, erythromycin, clindamycin, penicillin G, and vancomycin are enhanced when nanosilver is combined with them. Nanosilver may be a key component in the treatment of AIDS patients, according to a 2015 study by Suganya and colleagues. The scientists examined the antibacterial properties of biologically produced silver nanoparticles against strains derived from HIV-positive patients. Spirulina was utilized in the nanosilver synthesis. They have been changed and improved physically through the use of these particulate systems [179].

Additionally to being used to make bandages, dressings, and surgical masks, nanosilver also serves as an antibacterial agent. Additionally, as an antibacterial agent, nanosilver is used in the production of bandages, dressings, and surgical masks. Due to the gradual release of silver ions, prostheses and medical equipment with a nanosilver coating guarantee long-lasting antibacterial activity [180, 181].Copper oxide nanoparticles actively fight the influenza A virus, SARS virus, and other hospital-acquired illnesses [182].

On that scale, matter demonstrates entirely novel and unexpected qualities that obfuscate the usual distinctions between technical and scientific professions. Nanotechnology-based solutions are frequently employed in the medical field. The most significant instances of metal nanoparticle use in pharmacology, cancer treatment, and stomatology have been described in this paper [183]. By definition, nanobiotechnology is a multi-strategic method that combines nanotechnology and biotechnology to adjust the properties of therapeutic agents as the emphasis shifts from basic biological research to clinical applications. such as the targeted administration of therapies via nanoparticles. Nanoparticles have some peculiar properties, as a result, they can effectively diagnose and/or cure a variety of diseases, including cancer, by carefully adjusting their size, morphology, and surface properties. In addition, methods for better achieving therapeutic objectives rely on "responsive" nanomaterials that release the active components in response to specific stimuli, such as pH, redox potential, temperature, enzymes, or other variables that depend on external stimuli [184].

Green-synthesized nanoparticles (NPs) have demonstrated broad-spectrum utility across major biomedical applications such as antimicrobial, anticancer, wound healing, and targeted drug delivery. For example, silver nanoparticles (AgNPs) synthesized using *Azadirachta indica* or *Eclipta alba* extracts have shown minimum inhibitory concentrations (MICs) ranging from 3 to 10 µg/mL against *Escherichia coli* and *Staphylococcus aureus*, performing comparably or even better than their chemically synthesized counterparts [184, 186]. In anticancer evaluations, plant-derived ZnO and AuNPs showed selective cytotoxic effects with IC₅₀ values between 12 and 25 µg/mL against MCF-7 and A549 cancer cell lines, while sparing normal fibroblasts, indicating a high therapeutic index [187, 188]. In wound healing, AgNPs derived from green synthesis accelerated re-epithelialization and collagen deposition in rodent models, demonstrating faster closure and reduced infection rates [189].

Compared to chemically synthesized NPs, green NPs generally show improved biocompatibility, biodegradability, and lower in vitro cytotoxicity, due to the presence of surface-bound phytochemicals like polyphenols and terpenoids that act as natural capping and reducing agents [190, 191]. However, some in vivo toxicity concerns have been raised at higher dosages (>50 mg/kg), where hepatic or renal stress was noted in murine models [192]. In addition to in vitro assessments, several in vivo studies have reported favorable biosafety profiles for plant-derived nanoparticles. Long-term exposure studies in rodent models have shown that green-synthesized silver and zinc oxide nanoparticles, administered at therapeutic doses (<20 mg/kg), did not induce significant histopathological changes in major organs such as liver, kidney, or spleen over observation periods of 28–90 days. These findings suggest that phytochemical capping may mitigate systemic toxicity and improve in vivo tolerability, although comprehensive chronic toxicity and biodistribution studies remain limited and warrant further investigation. Additional challenges include inconsistencies in synthesis protocols, limited scale-up success, and lack of phytochemical standardization, which hinder reproducibility and regulatory approval. Although no plant-based nanoparticles have yet reached large-scale clinical trials, some AgNP formulations—such as gels and antimicrobial sprays—have entered preclinical and GMP-compliant pilot production for pharmaceutical applications [193, 194].

Although green-synthesized nanoparticles have found utility in diverse domains such as agriculture, textiles, and environmental remediation, only those applications with direct relevance to biomedical contexts are discussed herein. Specifically, recent studies have demonstrated that metal nanoparticles like silver (AgNPs) and zinc oxide (ZnO NPs), when integrated into medical textiles and implant coatings, provide robust antimicrobial barriers—significantly reducing risks of postoperative infections and biofilm formation on surgical implants [195, 196]. These coatings are especially crucial for orthopedic and dental implants, where microbial colonization is a leading cause of implant failure [197].

In the textile domain, biomedical relevance emerges when fabrics are modified with plant-derived nanoparticles to manufacture wound dressings or surgical gowns with sustained antimicrobial activity. For instance, silver nanoparticles synthesized from *Azadirachta indica* have been embedded in cotton dressings to inhibit *S. aureus* and *E. coli* colonization, illustrating the cross-section of textile and biomedical application [198]. Likewise, biodegradable polymer nanocomposites integrated with ZnO NPs from *Eclipta alba* are being explored for controlled drug-releasing wound patches, combining flexibility with therapeutic functionality [199].

The goal of contemporary healthcare is to raise patients' chances of survival and enhance their quality of life. To function as naturally as possible in the human body, implantable materials must be developed. Reliability and safety in usage are the essential requirements for implants comprised of artificial materials. They must not cause cancer, be toxic to living things, be inert to living tissues, be sufficiently mechanically robust, and be immune to the impacts of the internal environment of the body. [200]. Implant-associated infection is a common postoperative consequence of orthopaedic surgery that commonly results in patients experiencing agony, financial difficulty, and even death [201].

# 5.1 Dental Implants and Stress Distribution

Extensive research has demonstrated that restorative materials with a high elastic modulus, such as titanium, zirconia, and certain advanced ceramics, effectively reduce stress on the abutment, dental implant, and surrounding peri-implant bone during functional loading. These stiffer materials help distribute occlusal forces more evenly, minimizing stress concentrations that could lead to implant failure or bone resorption. Proper selection of restorative materials is essential, as it directly influences the implant’s ability to withstand excessive biting forces and parafunctional habits like bruxism. Alongside careful treatment planning and a thorough understanding of implant biomechanics, choosing materials with appropriate mechanical properties plays a crucial role in preventing complications. Since bone behavior is inversely related to load magnitude, materials that reduce excessive stress help preserve bone tissue and promote long-term implant success. Overall, combining suitable materials with meticulous clinical protocols significantly lowers the risk of implant failure and enhances patient outcomes [202–205].

Recently, plant-derived nanoparticles have been explored as surface modifiers for dental implants to enhance antibacterial activity and osseointegration. Green-synthesized silver and zirconium nanoparticles, capped with bioactive phytochemicals, have been incorporated into titanium and zirconia implant coatings to reduce peri-implant bacterial colonization while maintaining favorable stress distribution and mechanical integrity. Such biofunctionalized coatings offer a promising strategy to combine mechanical optimization with infection resistance in dental implant systems.

# 5.2. Metal implants

Titanium, its alloys, specific grades of stainless steel, and other conventional metals have been used as implants in medicine. The outstanding plastic properties, great chemical stability, and biocompatibility of titanium and its alloys make them the perfect materials for making load-bearing implants. Titanium and its alloys are also known for having high strength [206]. The insufficiently high wear and corrosion resistance, high coefficient of friction, and low bioactivity of Ti alloys, on the other hand, along with their high coefficient of friction indicates the need for additional surface modification. Additionally, abrasion of the implant that is too strong and the entry of metal ions into the bodily environment might decrease the implant's fixation and cause a hazardous reaction. Ti and its Ti64 alloy have been employed as biomaterials for implants since the 1950s, although their composition and surface characteristics are constantly changing. Ongoing research is being done on how thermomechanical processing affects the properties of titanium alloys.More research into developing new shape-memory, low Young's modulus titanium alloys [207, 208]. The danger of premature failure is higher for any material with Young's modulus close to that of bone. A porous metal implant would be the most effective technique to create a synthetic permanent replacement with bone's Young's modulus in this regard. These porous networks are adaptable to the host bone's mechanical properties, and their porous design encourages the host bone's fusion with it [209].

# 5.3 Ceramic implants

An additional type of implant material is ceramic, which is produced by sintering clays and clay mixtures with mineral additions, metal oxides, and other inorganic substances. In the late 1960s, interest in using ceramics for biomedical applications first emerged as a metal-free option with improved biocompatibility [210-214]. Bioceramic materials come in the following varieties depending on how the body reacts to the implant:The use of bioinert ceramics in bone prosthesis is constrained by several challenges despite the evident advantages of ceramic materials in terms of biochemical compatibility with the body when compared to metals and polymers currently employed for the restoration of the musculoskeletal system. Since it avoids the mechanical loads that lead to the resorption of the bone tissue surrounding the implant and its eventual loss, the low durability of such ceramics is a serious drawback. Recently, zirconium prosthetic implant failures have been recorded as a result of the implants' fast crack propagation. Research is now being done on a new generation [215]. Regarding stable or bioinert ceramics, the topic of altering their surfaces to enhance the process of the implant's osteointegration with the surrounding bone tissue is brought up. Given their inertia, giving the material antibacterial qualities to prevent adherence and multiplication of pathogenic germs is a requirement for their effective use. Modification techniques include spraying bioactive components, changing the surface topography and porosity in the area that will come into touch with bone tissue, and others. Making composite materials with new qualities from ceramics is an alternate method of altering them. Recent studies describe the use of laser ablation technology to create novel ZrO_2_ surfaces functionalized with Au nanoparticles or Ag microparticles. Friction testing showed that neither functionalized surface integrity was impaired after implant implantation, and the ZrO_2_ surface was uniformly distributed with particles. This led to the new idea of creating an antibacterial surface that combines functionalization at the nano- and micro-scales. A new class of therapeutic ceramic materials with properties that are superior to those of its current analogues will be created as research in this area moves further. [216].

# 5.4 Carbon nanostructured implants (CNI)

The biomedical field has identified carbon nanostructured implants (CNIs) as a remarkable clinical advance because of their green synthesis production approach alongside their remarkable biological adaptability [217]. Implants use engineering properties of carbon nanomaterials with extensive surface area and mechanical stability and electric current capabilities to optimize integration with tissue structures [218, 219]. Plants extracts and microorganisms in green synthesis reduce manufacturing toxic waste while creating more compatible materials which lower the body's immune reactions [220]. The specific design of CNIs helps tissue regeneration while guiding cell adhesion at implant sites because it enables direct delivery of treatment agents which results in better healing together with reduced infection risks [221, 222]. The flexible nature of CNIs enables their use in orthopedic, dental and cardiovascular implants which require enduring stability together with perfect biological integration. Sustainable nanotechnology development of green-synthesized CNIs creates opportunities for medical device revolution by providing safer substitute materials that match environmental commitments and patient safety protocols [223]. **Table 3** provides a comparative overview of the biological activities of green-synthesized silver and gold nanoparticles. Overall, AgNPs demonstrated superior antibacterial efficacy, exhibiting lower minimum inhibitory concentration (MIC) values against both Gram-positive and Gram-negative bacterial strains, particularly *Pseudomonas aeruginosa*.

# Table 3. Summary of biological activities of green-synthesized silver and gold nanoparticles [224].

| **Nanoparticle** | **Biological assay** | **Test model** | **Quantitative result** | **Key observation** |
| --- | --- | --- | --- | --- |
| AgNPs | Antibacterial (MIC) | MRSA, MSSA, K. pneumoniae | 250 µg/mL | Higher antibacterial potency than AuNPs |
| AgNPs | Antibacterial (MIC) | P. aeruginosa | 125 µg/mL | Most sensitive bacterial strain |
| AuNPs | Antibacterial (MIC) | MRSA, K. pneumoniae | 1000 µg/mL | Lower antibacterial efficacy |
| AuNPs | Antibacterial (MIC) | MSSA, P. aeruginosa | 500 µg/mL | Moderate inhibition |
| AgNPs | Cytotoxicity (IC50) | Vero cells | 693.68 µg/mL | Low toxicity to normal cells |
| AuNPs | Cytotoxicity (IC50) | Vero cells | 661.24 µg/mL | Comparable biocompatibility |
| AgNPs | Anticancer (IC50) | MCF-7 cells | 370.56 µg/mL | Higher anticancer potency |
| AuNPs | Anticancer (IC50) | MCF-7 cells | 394.79 µg/mL | Moderate anticancer activity |
| AgNPs | Antioxidant (DPPH IC50) | Free radical scavenging | 19.7 µg/mL | Strong antioxidant activity |
| AuNPs | Antioxidant (DPPH IC50) | Free radical scavenging | 194.0 µg/mL | Weaker antioxidant activity |

In cytotoxicity assessments using Vero cells, both AgNPs and AuNPs displayed relatively high IC₅₀ values, indicating low toxicity toward normal mammalian cells and favorable biocompatibility profiles. Notably, AgNPs showed enhanced anticancer activity against MCF-7 breast cancer cells compared to AuNPs, suggesting greater therapeutic potential. In addition, AgNPs exhibited significantly stronger antioxidant activity, as reflected by lower DPPH IC₅₀ values, whereas AuNPs showed comparatively weaker radical-scavenging capacity. Collectively, these findings highlight the metal-dependent variation in biological performance and underscore the enhanced multifunctional bioactivity of plant-derived AgNPs relative to AuNPs.

**6. Challenges and Future Perspectives**

Despite extensive progress in green synthesis of nanoparticles (NPs), several critical challenges continue to impede their clinical and industrial translation.

#### ****6.1 Regulatory and Industrialization Challenges****

A major bottleneck in the commercialization of plant-derived nanoparticles is the lack of standardized synthesis protocols. Factors such as variations in plant phytochemical profiles, seasonal fluctuations, and inconsistent extraction techniques result in reproducibility issues [225]. These inconsistencies make it difficult to meet regulatory requirements set by agencies like the FDA or EMA, which require stringent quality control, pharmacokinetic data, and toxicological assessments [226]. Moreover, while green synthesis is environmentally friendly and cost-effective, its industrial scalability remains limited. Challenges include sourcing consistent raw plant material, maintaining nanoparticle uniformity during scale-up, and optimizing purification methods to meet Good Manufacturing Practice (GMP) standards. Few successful examples have moved beyond the lab, and those that have remain in early pilot or preclinical stages.

#### ****6.2 Toxicological and Pharmacokinetic Gaps****

Another significant barrier is the lack of comprehensive toxicological profiling. Although many studies report favorable **in vitro** biocompatibility, **in vivo** data on long-term toxicity, immunogenicity, and organ-specific accumulation are scarce [227]. Similarly, pharmacokinetic studies—critical for determining dosing, metabolism, and excretion—are limited or entirely absent in most publications. The immune response to plant-based NPs remains poorly understood. There is an urgent need for systematic investigations into whether these NPs trigger pro-inflammatory reactions, cytokine release, or hypersensitivity, especially in biomedical applications like drug delivery [228].

#### ****6.3 Future Research Directions****

To advance the clinical and industrial application of green-synthesized nanoparticles, future research should prioritize the standardization of synthesis protocols—including solvent type, pH, temperature, and extraction methods—to ensure inter-study reproducibility and meet regulatory standards [229]. Comprehensive pharmacokinetic and biodistribution studies in animal models are essential to predict behavior in human systems and guide therapeutic dosing [230]. Equally important is the investigation of long-term and dose-dependent toxicological effects under physiological conditions to ensure safety [231]. Detailed studies on immune interactions—such as cytokine profiling, complement activation and histopathological analysis—are needed to assess potential immunogenic responses [232, 233]. To facilitate scalable production, the development of bioreactor-based or automated synthesis platforms compliant with Good Manufacturing Practices (GMP) should be pursued [234, 235]. Additionally, creating centralized, publicly accessible databases compiling synthesis methods, biological activity, and safety data would support future meta-analyses, improve transparency, and assist in regulatory approval processes [236]. Addressing these priorities will be crucial for translating plant-based nanotechnology into practical, safe, and scalable solutions across medicine, agriculture, and environmental sectors.

7. Conclusion

The use of plant-derived nanoparticles offers a promising route for the creation of advanced nanomaterials with a range of uses. Numerous benefits, such as affordability, scalability, and environmental sustainability, are provided by the green synthesis techniques used in their production. Insights into the structural and functional characteristics of plant-based nanoparticles can be gained from the characterization methods used to evaluate their physicochemical characteristics. Applications for nanoparticles made from plants include catalysis, energy storage, environmental remediation, healthcare, and agriculture. Due to their improved biocompatibility and capacity for targeted delivery, these nanoparticles have shown great promise in the healthcare industry as drug delivery systems, antimicrobial agents, and anticancer agents. Plant-based nanoparticles have demonstrated potential in agriculture as nanofertilizers, pesticides, and growth regulators, enhancing crop sustainability and minimizing environmental impact. Additionally, their uses in environmental cleanup, catalysis, and energy storage demonstrate their adaptability and potential to address important global issues. Despite the substantial advancements made in this area, difficulties still exist. Future research must focus on standardizing synthesis procedures, improving the stability and reproducibility of nanoparticles, and thoroughly analyzing their long-term effects on living systems. To enable their widespread application, plant-based nanoparticles' commercial viability and scalability need to be further investigated.

**Date Availability**

The authors confirm that the data supporting the findings of this study are available within the article.

**Acknowledgement**

We would like to express our deep gratitude to Dreamers Lab for their invaluable support and guidance, which have played a pivotal role in shaping us into the skilled researchers we aspired to become. Additionally, we are indebted to Dreamers Lab for their ongoing technical assistance, which has been instrumental in the success of our work.

**Conflict of Interest**

This manuscript declares no conflicts of interest.

**Funding Information**

This manuscript received no funding.

**Author Contribution**

**Md Hosne Mobarak** – Resources, Writing, Visualization**; Md. Zobair Al Mahmud**– Reviewing, Writing, Visualization**; Amran Hossain** –Editing, Writing, Visualization**; Md. Arman Hossain Abir** – Writing, Visualization**; Nayem Hossain** – Conceptualization; **Mohammad Asaduzzaman Chowdhury** – Resources, Supervision, Visualization, Validation.

**Statement of Usage of Artificial Intelligence**

We used AI tools (ChatGPT, Stealth Writer, and QuillBot) solely for language enhancement purposes, such as grammar correction and sentence rephrasing. No data, results, or scientific content were generated by AI. All analyses, interpretations, and conclusions are the authors' own.

References

1. Hanemann, T., & Szabó, D. V. (2010). Polymer-Nanoparticle Composites: From Synthesis to Modern Applications. *Materials*, *3*(6), 3468-3517. https://doi.org/10.3390/ma3063468
2. Hossain, N., Mobarak, M. H., Hossain, A., Khan, F., Mim, J. J., & Chowdhury, M. A. (2023). Advances of plant and biomass extracted zirconium nanoparticles in dental implant application. *Heliyon*, *9*(5), e15973. <https://doi.org/10.1016/j.heliyon.2023.e15973>
3. Munir, H., Bilal, M., Mulla, S. I., Abbas Khan, H., & Iqbal, H. M. (2021). Plant-mediated green synthesis of nanoparticles. In *Advances in green synthesis: Avenues and sustainability* (pp. 75-89). Cham: Springer International Publishing.
4. Ahmed, S., Chaudhry, S. A., & Ikram, S. (2020). A review on biogenic synthesis of ZnO nanoparticles using plant extracts and microbes: A prospect towards green chemistry. Journal of Photochemistry and Photobiology B: Biology, 166, 272–284. <https://doi.org/10.1016/j.jphotobiol.2016.12.011>
5. Akintelu, S. A., & Folorunso, A. S. (2020). A review on green synthesis of zinc oxide nanoparticles using plant extracts and its biomedical applications. BioNanoScience, 10(4), 848–863. https://doi.org/10.1007/s12668-020-00739-4
6. Hano, C., & Abbasi, B. H. (2022). Plant-based green synthesis of nanoparticles: Production, characterization and applications. Biomolecules, 12(1), 31. <https://doi.org/10.3390/biom12010031>
7. Ahmed, S., Chaudhry, S. A., & Ikram, S. (2017). A review on biogenic synthesis of ZnO nanoparticles using plant extracts and microbes: A prospect towards green chemistry. *Journal of Photochemistry and Photobiology B: Biology*, *166*, 272-284. <https://doi.org/10.1016/j.jphotobiol.2016.12.011>
8. Tundo, P., Anastas, P., Black, D., Breen, J., Collins, T., Memoli, S., Miyamoto, J., Polyakoff, M. & Tumas, W. (2000). Synthetic pathways and processes in green chemistry. Introductory overview. *Pure and Applied Chemistry*, *72*(7), 1207-1228. <https://doi.org/10.1351/pac200072071207>
9. Hano, C., & Abbasi, B. H. (2022). Plant-Based Green Synthesis of Nanoparticles: Production, Characterization and Applications. *Biomolecules*, *12*(1), 31. <https://doi.org/10.3390/biom12010031>
10. Tosif, M. M., Najda, A., Bains, A., Kaushik, R., Dhull, S. B., & Chawla, P. (2021). A Comprehensive Review on Plant-Derived Mucilage: Characterization, Functional Properties, Applications, and Its Utilization for Nanocarrier Fabrication. *Polymers*, *13*(7), 1066. <https://doi.org/10.3390/polym13071066>
11. Kuppusamy, P., Yusoff, M. M., Maniam, G. P., & Govindan, N. (2016). Biosynthesis of metallic nanoparticles using plant derivatives and their new avenues in pharmacological applications – An updated report. *Saudi Pharmaceutical Journal*, *24*(4), 473-484. <https://doi.org/10.1016/j.jsps.2014.11.013>
12. Prateek Mathur, Swati Jha, Suman Ramteke & N. K. Jain (2018) Pharmaceutical aspects of silver nanoparticles, Artificial Cells, Nanomedicine, and Biotechnology, 46:sup1, 115-126, DOI: 10.1080/21691401.2017.1414825
13. Gunti, L., Dass, R. S., & Kalagatur, N. K. (2019). Phytofabrication of Selenium Nanoparticles From Emblica officinalis Fruit Extract and Exploring Its Biopotential Applications: Antioxidant, Antimicrobial, and Biocompatibility. *Frontiers in Microbiology*, *10*. <https://doi.org/10.3389/fmicb.2019.00931>
14. Zheng, X., Zhang, P., Fu, Z., Meng, S., Dai, L., & Yang, H. (2021). Applications of nanomaterials in tissue engineering. *RSC Advances*, *11*(31), 19041–19058. <https://doi.org/10.1039/d1ra01849c>
15. Prasad, R. (2014). Synthesis of silver nanoparticles in photosynthetic plants. *Journal of Nanoparticles*, *2014*.
16. Boenink, M., Swierstra, T., & Stemerding, D. (2010). Anticipating the interaction between technology and morality: A scenario study of experimenting with humans in bionanotechnology. Studies in ethics, law, and technology, 4(2).
17. Shah, S. S., Shaikh, M. N., Khan, M. Y., Alfasane, M. A., Rahman, M. M., & Aziz, M. A. (2021). Present status and future prospects of jute in nanotechnology: A review. The Chemical Record, 21(7), 1631-1665.
18. Rabbani, M. M., Ahmed, I., & Park, S. J. (2016). Application of nanotechnology to remediate contaminated soils. Environmental remediation technologies for metal-contaminated soils, 219-229.
19. Rasmussen, J. W., Martinez, E., Louka, P., & Wingett, D. G. (2010). Zinc oxide nanoparticles for selective destruction of tumor cells and potential for drug delivery applications. Expert opinion on drug delivery, 7(9), 1063-1077.
20. Pantidos, N., & Horsfall, L. E. (2014). Biological synthesis of metallic nanoparticles by bacteria, fungi and plants. Journal of Nanomedicine & Nanotechnology, 5(5), 1.
21. Muddapur, U. M., Alshehri, S., Ghoneim, M. M., Mahnashi, M. H., Alshahrani, M. A., Khan, A. A., Iqubal, S. M., Bahafi, A., More, S. S., Shaikh, I. A., Mannasaheb, B. A., Othman, N., Maqbul, M. S., & Ahmad, M. Z. (2022). Plant-Based Synthesis of Gold Nanoparticles and Theranostic Applications: A Review. *Molecules*, *27*(4), 1391. <https://doi.org/10.3390/molecules27041391>
22. Singh, P., Kim, Y., Zhang, D., & Yang, D. (2016). Biological Synthesis of Nanoparticles from Plants and Microorganisms. *Trends in Biotechnology*, *34*(7), 588-599. <https://doi.org/10.1016/j.tibtech.2016.02.006>
23. Das, R. K., Pachapur, V. L., Lonappan, L., Naghdi, M., Pulicharla, R., Maiti, S., ...& Brar, S. K. (2017). Biological synthesis of metallic nanoparticles: plants, animals and microbial aspects. Nanotechnology for Environmental Engineering, 2, 1-21.
24. Reverberi, A., Vocciante, M., Lunghi, E., Pietrelli, L., & Fabiano, B. (2017). New trends in the synthesis of nanoparticles by green methods. Chemical Engineering Transactions, 61, 667-672.
25. Mohammed, A. E., Al-Qahtani, A., Al-Mutairi, A., Al-Shamri, B., & Aabed, K. (2018). Antibacterial and cytotoxic potential of biosynthesized silver nanoparticles by some plant extracts. Nanomaterials, 8(6), 382.
26. Pavela, R. (2016). History, presence and perspective of using plant extracts as commercial botanical insecticides and farm products for protection against insects–a review. Plant Protection Science, 52(4), 229-241.
27. Bhadoriya, S. S., Mangal, A., Madoriya, N., & Dixit, P. (2011). Bioavailability and bioactivity enhancement of herbal drugs by “Nanotechnology”: a review. J Curr Pharm Res, 8(1), 1-7.
28. Adelere, I. A., & Lateef, A. (2016). A novel approach to the green synthesis of metallic nanoparticles: the use of agro-wastes, enzymes, and pigments. *Nanotechnology Reviews*, *5*(6), 567-587.
29. Patil, S., & Chandrasekaran, R. (2020). Biogenic nanoparticles: A comprehensive perspective in synthesis, characterization, application and its challenges. *Journal of Genetic Engineering and Biotechnology*, *18*(1), 1-23. <https://doi.org/10.1186/s43141-020-00081-3>
30. Venkat Kumar, S., & Rajeshkumar, S. (2018). Plant-Based Synthesis of Nanoparticles and Their Impact. *Nanomaterials in Plants, Algae, and Microorganisms*, 33-57. <https://doi.org/10.1016/B978-0-12-811487-2.00002-5>
31. Li, S., Shen, Y., Xie, A., Yu, X., Qiu, L., Zhang, L., & Zhang, Q. (2007). Green synthesis of silver nanoparticles using Capsicum annuum L. extractElectronic supplementary information (ESI) available: Details of the extracting process of Capsicum annuum L., figures of N1s and O1s core level spectra, and the frequencies and the percentages of the secondary structures of Capsicum annuum L. proteins. See.
32. Armendariz, V., Herrera, I., Peralta-Videa, J. R., Jose-Yacaman, M., Troiani, H., Santiago, P., & Gardea-Torresdey, J. L. (2004). Size controlled gold nanoparticle formation by Avena sativa biomass: use of plants in nanobiotechnology. Journal of nanoparticle research, 6, 377-382.
33. Sathishkumar, M., Sneha, K., Won, S. W., Cho, C. W., Kim, S., & Yun, Y. S. (2009). Cinnamon zeylanicum bark extract and powder mediated green synthesis of nano-crystalline silver particles and its bactericidal activity. Colloids and surfaces B: Biointerfaces, 73(2), 332-338.
34. Salem, S. S. (2023). A mini review on green nanotechnology and its development in biological effects. Archives of Microbiology, 205(4), 128.
35. Mude, N., Ingle, A., Gade, A., & Rai, M. (2009). Synthesis of silver nanoparticles using callus extract of Carica papaya—a first report. Journal of Plant Biochemistry and Biotechnology, 18, 83-86.
36. Bar, H., Bhui, D. K., Sahoo, G. P., Sarkar, P., De, S. P., & Misra, A. (2009). Green synthesis of silver nanoparticles using latex of Jatropha curcas. Colloids and surfaces A: Physicochemical and engineering aspects, 339(1-3), 134-139.
37. Giardina, E., Spinella, A., & Novelli, G. (2011). Past, present and future of forensic DNA typing. Nanomedicine, 6(2), 257-270.
38. Sharma, V. K., Yngard, R. A., & Lin, Y. (2009). Silver nanoparticles: green synthesis and their antimicrobial activities. Advances in colloid and interface science, 145(1-2), 83-96.
39. Rastogi, A., Singh, P., Haraz, F. A., & Barhoum, A. (2018). Biological synthesis of nanoparticles: An environmentally benign approach. *Fundamentals of Nanoparticles*, 571-604. <https://doi.org/10.1016/B978-0-323-51255-8.00023-9>
40. Ahmad, A., Senapati, S., Khan, M.I., Kumar, R., Sastry, M., Langmuir, 19 (2003), 3550–3553.
41. Gericke, M., & Pinches, A. (2006). Biological synthesis of metal nanoparticles. *Hydrometallurgy*, *83*(1-4), 132-140. <https://doi.org/10.1016/j.hydromet.2006.03.019>
42. El-Khawaga, A. M., Zidan, A., & Abd El-Mageed, A. I. (2023). Preparation methods of different nanomaterials for various potential applications: A Review. Journal of Molecular Structure, 1281, 135148.
43. Keat, C. L., Aziz, A., Eid, A. M., & Elmarzugi, N. A. (2015). Biosynthesis of nanoparticles and silver nanoparticles. *Bioresources and Bioprocessing*, *2*(1), 1-11. <https://doi.org/10.1186/s40643-015-0076-2>
44. Kumar, S., Basumatary, I. B., Sudhani, H. P., Bajpai, V. K., Chen, L., Shukla, S., & Mukherjee, A. (2021). Plant extract mediated silver nanoparticles and their applications as antimicrobials and in sustainable food packaging: A state-of-the-art review. *Trends in Food Science & Technology*, *112*, 651-666. <https://doi.org/10.1016/j.tifs.2021.04.031>
45. Usman, K. A. S., Maina, J. W., Seyedin, S., Conato, M. T., Payawan Jr, L. M., Dumée, L. F., & Razal, J. M. (2020). Downsizing metal–organic frameworks by bottom-up and top-down methods. NPG Asia Materials, 12(1), 58.
46. Facibeni, A. (2023). Silver Nanoparticles: Synthesis, Properties, and Applications. CRC Press.
47. Khalaj, M., Kamali, M., Costa, M. E. V., & Capela, I. (2020). Green synthesis of nanomaterials - A scientometric assessment. *Journal of Cleaner Production*, *267*, 122036. <https://doi.org/10.1016/j.jclepro.2020.122036>
48. Kumar, S., Kumar, B., Sehgal, R., Wani, M. F., Kumar, D., Sharma, M. D., ...& Kumar, V. (2023). Advantages and Disadvantages of Metal Nanoparticles. In Nanoparticles Reinforced Metal Nanocomposites: Mechanical Performance and Durability (pp. 209-235). Singapore: Springer Nature Singapore.
49. Hussein, H. (2023). The state of the art of nanomaterials and its applications in energy saving. Bulletin of the National Research Centre, 47(1), 1-22.
50. Ahmed, M. (2020). Nanomaterial synthesis. *Polymer Science and Nanotechnology*, 361-399. <https://doi.org/10.1016/B978-0-12-816806-6.00016-9>
51. Aseel, D. G., Behiry, S. I., & Abdelkhalek, A. (2023). Green and Cost-Effective Nanomaterials Synthesis from Desert Plants and Their Applications. In Secondary Metabolites Based Green Synthesis of Nanomaterials and Their Applications (pp. 327-357). Singapore: Springer Nature Singapore.
52. Akter, S., Lee, S. Y., Siddiqi, M. Z., Balusamy, S. R., Ashrafudoulla, M., Rupa, E. J., & Huq, M. A. (2020). Ecofriendly synthesis of silver nanoparticles by Terrabacter humi sp. nov. and their antibacterial application against antibiotic-resistant pathogens. International Journal of Molecular Sciences, 21(24), 9746.
53. Du, J., Singh, H., & Yi, T. H. (2016). Antibacterial, anti-biofilm and anticancer potentials of green synthesized silver nanoparticles using benzoin gum (Styrax benzoin) extract. Bioprocess and biosystems engineering, 39, 1923-1931.
54. Vigneshwaran, N., Kathe, A. A., Varadarajan, P. V., Nachane, R. P., & Balasubramanya, R. H. (2007). Silver− protein (core− shell) nanoparticle production using spent mushroom substrate. Langmuir, 23(13), 7113-7117.
55. Kavitha, K. S., Baker, S., Rakshith, D., Kavitha, H. U., Yashwantha Rao, H. C., Harini, B. P., & Satish, S. (2013). Plants as green source towards synthesis of nanoparticles. Int Res J Biol Sci, 2(6), 66-76.
56. Iravani, S. (2011). Green synthesis of metal nanoparticles using plants. Green chemistry, 13(10), 2638-2650.
57. Park, Y., Hong, Y. N., Weyers, A., Kim, Y. S., & Linhardt, R. J. (2011). Polysaccharides and phytochemicals: a natural reservoir for the green synthesis of gold and silver nanoparticles. IET nanobiotechnology, 5(3), 69-78.
58. Zahir, A. A., Bagavan, A., Kamaraj, C., Elango, G., & Rahuman, A. A. (2015). Green synthesis of silver and titanium dioxide nanoparticles using Euphorbia prostrata extract shows shift from apoptosis to G0/G1 arrest followed by necrotic cell death in Leishmania donovani. Antimicrobial Agents and Chemotherapy, 59(8), 4782–4799. https://doi.org/10.1128/AAC.00111-15
59. Momeni, S., Nabipour, I., & Arami, M. (2015). A simple green synthesis of palladium nanoparticles with Sargassum alga and their electrocatalytic activities towards hydrogen peroxide. Applied Biochemistry and Biotechnology, 176(7), 1937–1949. https://doi.org/10.1007/s12010-015-1674-7
60. Nasrollahzadeh, M., Sajadi, S. M., Rostami-Vartooni, A., & Bagherzadeh, M. (2015). Green synthesis of copper nanoparticles using Ginkgo biloba L. leaf extract and their catalytic activity for the Huisgen [3+2] cycloaddition of azides and alkynes at room temperature. Journal of Colloid and Interface Science, 457, 141–147. https://doi.org/10.1016/j.jcis.2015.07.010
61. Yugay, Y., Rusapetova, T., Mashtalyar, D., Grigorchuk, V., Vasyutkina, E., Kudinova, O., ... & Shkryl, Y. (2021). Biomimetic synthesis of functional silver nanoparticles using hairy roots of Panax ginseng for wheat pathogenic fungi treatment. Colloids and Surfaces B: Biointerfaces, 207, 112031. https://doi.org/10.1016/j.colsurfb.2021.112031
62. Singh, P., Kim, Y. J., Zhang, D., & Yang, D. C. (2015). Biogenic silver and gold nanoparticles synthesised using red ginseng root extract, and their applications. Artificial Cells, Nanomedicine, and Biotechnology. Advance online publication. <https://doi.org/10.3109/21691401.2015.1008514>
63. Elango, G., Roopan, S. M., & Rahuman, A. A. (2015). Green synthesis, spectroscopic investigation and photocatalytic activity of lead nanoparticles. Spectrochimica Acta Part A: Molecular and Biomolecular Spectroscopy, 139, 367–373. https://doi.org/10.1016/j.saa.2014.12.086
64. Zhou, G. J., Han, R., Zhou, Y., & Zhang, J. (2014). Biosynthesis of CdS nanoparticles in banana peel extract. Journal of Nanoscience and Nanotechnology, 14(6), 4437–4442. https://doi.org/10.1166/jnn.2014.7993
65. Shende, S., Ingle, A. P., Gade, A., & Rai, M. (2015). Green synthesis of copper nanoparticles by Citrus medica Linn. (Idilimbu) juice and its antimicrobial activity. World Journal of Microbiology and Biotechnology, 31(6), 865–873. https://doi.org/10.1007/s11274-015-1830-3
66. Ærøe Hyllested, J., Espina Palanco, M., Hernández-Garrido, J. C., Blanco-Andujar, C., & Nogués, C. (2015). Green preparation and spectroscopic characterization of plasmonic silver nanoparticles using fruits as reducing agents. Beilstein Journal of Nanotechnology, 6, 293–299. https://doi.org/10.3762/bjnano.6.28
67. Naseem, T., & Farrukh, M. A. (2015). Antibacterial activity of green synthesis of iron nanoparticles using Lawsonia inermis and Gardenia jasminoides leaves extract. Journal of Chemistry, 2015, 912342. https://doi.org/10.1155/2015/912342
68. Poopathi, S., De Britto, L. J., & Ragavendran, C. (2015). Synthesis of silver nanoparticles from Azadirachta indica–a most effective method for mosquito control. Environmental Science and Pollution Research International, 22(4), 2956–2963. https://doi.org/10.1007/s11356-014-3487-4
69. Amooaghaie, R., Saeri, M. R., & Azizi, M. (2015). Synthesis, characterization and biocompatibility of silver nanoparticles synthesised from Nigella sativa leaf extract in comparison with chemical silver nanoparticles. Ecotoxicology and Environmental Safety, 120, 400–408. https://doi.org/10.1016/j.ecoenv.2015.06.025
70. Kalaiselvi, A., Ravikumar, R., Rajiv, P., & Sankaranarayanan, K. (2015). Synthesis and characterization of palladium nanoparticles using Catharanthus roseus leaf extract and its application in the photo-catalytic degradation. Spectrochimica Acta Part A: Molecular and Biomolecular Spectroscopy, 135, 116–119. https://doi.org/10.1016/j.saa.2014.06.137
71. Sadeghi, B., Garmaroudi, F. S., Hashemi, M., Nezhad, H. R., Nasrollahzadeh, M., & Ardalan, S. (2015). Facile green synthesis of silver nanoparticles using seed aqueous extract of Pistacia atlantica and its antibacterial activity. Spectrochimica Acta Part A: Molecular and Biomolecular Spectroscopy, 134, 326–332. https://doi.org/10.1016/j.saa.2014.06.134
72. Gogoi, N., Barua, R., Khan, R., & Devi, D. (2015). Green synthesis and characterization of silver nanoparticles using alcoholic flower extract of Nyctanthes arbortristis and in vitro investigation of their antibacterial and cytotoxic activities. Materials Science and Engineering: C, 46, 463–469. https://doi.org/10.1016/j.msec.2014.10.050
73. Suresh, D., Nethravathi, P. C., Udayabhanu, Nagaraju, G., & Sharma, S. C. (2015). Artocarpus gomezianus aided green synthesis of ZnO nanoparticles: Luminescence, photocatalytic and antioxidant properties. Spectrochimica Acta Part A: Molecular and Biomolecular Spectroscopy, 141, 128–134. https://doi.org/10.1016/j.saa.2014.12.067
74. Murugan, K., Jaffar Ali, H. A., Panneerselvam, C., Roni, M., & Subramaniam, J. (2015). Cymbopogon citratus-synthesised gold nanoparticles boost the predation efficiency of copepod Mesocyclops aspericornis against malaria and dengue mosquitoes. Experimental Parasitology, 153, 129–138. https://doi.org/10.1016/j.exppara.2015.03.018
75. Siddiqi, K. S., & Husen, A. (2020). Current status of plant metabolite-based fabrication of copper/copper oxide nanoparticles and their applications: A review. Biomaterials Research, 24(1), 11. https://doi.org/10.1186/s40824-020-00193-w
76. Saligedo, T. S., Muleta, G. G., Tsega, T. W., & Tadele, K. T. (2023). Green Synthesis of Copper Oxide Nanoparticles Using Eichhornia Crassipes Leaf Extract, its Antibacterial and Photocatalytic Activities. Current Nanomaterials, 8(1), 58-68.
77. Husen, A. (Ed.). (2023). *Secondary Metabolites Based Green Synthesis of Nanomaterials and Their Applications*. Springer Nature.
78. Wanche Kojom, J. J., Bogning, C. Z., Lappa, E. L., Sonfack, C. S., Kuinze, A. N., Etamé-Loé, G., & Dongmo, A. B. (2023). Antioxidant Properties and Vasorelaxant Mechanism of Aqueous Extract of Ricinodendron heudelotii (Euphorbiaceae). *BioMed Research International*, *2024*(1), 3435974. https://doi.org/10.1155/2024/3435974
79. Nasrollahzadeh, M., Sajadi, S. M., Rostami-Vartooni, A., & Hussin, S. M. (2016). Green synthesis of CuO nanoparticles using aqueous extract of *Thymus vulgaris* L. leaves and their catalytic performance for N-arylation of indoles and amines. *Journal of Colloid and Interface Science, 466*, 113–119. https://doi.org/10.1016/j.jcis.2015.12.015
80. Ghidan, A. Y., Al-Antary, T. M., & Awwad, A. M. (2016). Green synthesis of copper oxide nanoparticles using Punica granatum peels extract: Effect on green peach Aphid. *Environmental Nanotechnology, Monitoring & Management*, *6*, 95-98. https://doi.org/10.1016/j.enmm.2016.08.002
81. Geremew, A., Palmer, L., Johnson, A., Reeves, S., Brooks, N., & Carson, L. (2024). Multi-functional copper oxide nanoparticles synthesized using Lagerstroemia indica leaf extracts and their applications. Heliyon, 10(9).
82. Kumar, L., Mohan, L., Anand, R., & Bharadvaja, N. (2023). Chlorella minutissima-assisted silver nanoparticles synthesis and evaluation of its antibacterial activity. Systems Microbiology and Biomanufacturing, 1-10.
83. Yousaf, H., Mehmood, A., Ahmad, K. S., & Raffi, M. (2020). Green synthesis of silver nanoparticles and their applications as an alternative antibacterial and antioxidant agents. *Materials Science and Engineering: C*, *112*, 110901. <https://doi.org/10.1016/j.msec.2020.110901>
84. Habeeb Rahuman, H. B., Dhandapani, R., Narayanan, S., Palanivel, V., Paramasivam, R., Subbarayalu, R., ... & Muthupandian, S. (2022). Medicinal plants mediated the green synthesis of silver nanoparticles and their biomedical applications. *IET nanobiotechnology*, *16*(4), 115-144.
85. Abdelghany, T. M., Al-Rajhi, A. M., Al Abboud, M. A., Alawlaqi, M. M., Ganash Magdah, A., Helmy, E. A., & Mabrouk, A. S. (2018). Recent advances in green synthesis of silver nanoparticles and their applications: about future directions. A review. *BioNanoScience*, *8*(1), 5-16.
86. Goodsell, D. S. (2004). *Bionanotechnology: lessons from nature*. John Wiley & Sons.
87. Vera, J., Herrera, W., Hermosilla, E., Díaz, M., Parada, J., Seabra, A. B., ...& Rubilar, O. (2023). Antioxidant Activity as an Indicator of the Efficiency of Plant Extract-Mediated Synthesis of Zinc Oxide Nanoparticles. Antioxidants, 12(4), 784.
88. Jha, S., Rani, R., & Singh, S. (2023). Biogenic Zinc Oxide Nanoparticles and Their Biomedical Applications: A Review. Journal of Inorganic and Organometallic Polymers and Materials, 1-16.
89. Sutradhar, P., & Saha, M. (2017). Green synthesis of zinc oxide nanoparticles using tomato (Lycopersicon esculentum) extract and its photovoltaic application. Journal of Experimental Nanoscience, 11, 314–327. [https://doi.org/10.1080/17458080. 2015.1059504](https://doi.org/10.1080/17458080.%202015.1059504).
90. Singh, A. K., Pal, P., Gupta, V., Yadav, T. P., Gupta, V., & Singh, S. P. (2018). Green synthesis, characterization and antimicrobial activity of zinc oxide quantum dots using *Eclipta alba*. Materials Chemistry and Physics, 203, 40–48. https://doi.org/10.1016/j. matchemphys.2017.09.049.
91. Gupta, M., Tomar, R. S., Kaushik, S., Mishra, R. K., & Sharma, D. (2018). Effective antimicrobial activity of green ZnO nano particles of Catharanthus roseus. Frontiers in Microbiology, 9, 1–13. https://doi.org/10.3389/fmicb.2018.02030.
92. Singh, K., Singh, J., & Rawat, M. (2019). Green synthesis of zinc oxide nanoparticles using Punica Granatum leaf extract and its application towards photocatalytic degradation of Coomassie brilliant blue R-250 dye. SN Applied Sciences, 1, 1–8. https://doi.org/ 10.1007/s42452-019-0610-5.
93. Akintelu, S. A., & Folorunso, A. S. (2020). A review on green synthesis of zinc oxide nanoparticles using plant extracts and its biomedical applications. BioNanoScience, 10(4), 848-863.
94. Thema, F., Manikandan, E., Dhlamini, M., & Maaza, M. (2015). Green synthesis of ZnO nanoparticles via Agathosma betulina natural extract. *Materials Letters*, *161*, 124-127. https://doi.org/10.1016/j.matlet.2015.08.052
95. Gardea-Torresdey, J. L., Parsons, J. G., Gomez, E., Peralta-Videa, J., Troiani, H. E., Santiago, P., & Yacaman, M. J. (2002). Formation and growth of Au nanoparticles inside live alfalfa plants. Nano letters, 2(4), 397-401.
96. Seku, K., Hussaini, S. S., Reddy, M. R., Reddy, G. B., & Kumar, K. K. (2023). Fungal-mediated synthesis of gold nanoparticles and their biological applications. In Fungal Cell Factories for Sustainable Nanomaterials Productions and Agricultural Applications (pp. 23-58). Elsevier.
97. Haverkamp, R. G., Marshall, A. T., & van Agterveld, D. (2007). Pick your carats: Nanoparticles of gold–silver–copper alloy produced in vivo. *Journal of Nanoparticle Research, 9*(4), 697–700. https://doi.org/10.1007/s11051-006-9161-7
98. Keshavarzi, M., Davoodi, D., Pourseyedi, S., & Taghizadeh, S. (2018). The effects of three types of alfalfa plants (*Medicago sativa*) on the biosynthesis of gold nanoparticles: An insight into phytomining. *Gold Bulletin, 51*(2), 99–110. https://doi.org/10.1007/s13404-018-0229-6
99. Bali, R., & Harris, A. T. (2010). Biogenic synthesis of Au nanoparticles using vascular plants. Industrial & engineering chemistry research, 49(24), 12762-12772.
100. Bharadwaj, K. K., Rabha, B., Pati, S., Sarkar, T., Choudhury, B. K., Barman, A., Bhattacharjya, D., Srivastava, A., Baishya, D., Edinur, H. A., Abdul Kari, Z., & Mohd Noor, N. H. (2021). Green Synthesis of Gold Nanoparticles Using Plant Extracts as Beneficial Prospect for Cancer Theranostics. *Molecules*, *26*(21), 6389. https://doi.org/10.3390/molecules26216389
101. Lee, K. X., Shameli, K., Yew, Y. P., Teow, S. Y., Jahangirian, H., Rafiee-Moghaddam, R., & Webster, T. J. (2020). Recent developments in the facile bio-synthesis of gold nanoparticles (AuNPs) and their biomedical applications. International journal of nanomedicine, 275-300.
102. Shafey, A. M. E. (2020). Green synthesis of metal and metal oxide nanoparticles from plant leaf extracts and their applications: A review. Green Processing and Synthesis, 9(1), 304-339.
103. Bratovcic, A. (2019). Different applications of nanomaterials and their impact on the environment. SSRG International Journal of Material Science and Engineering, 5(1), 1-7.
104. Jafarzadeh, S., Nooshkam, M., Zargar, M., Garavand, F., Ghosh, S., Hadidi, M., & Forough, M. (2023). Green synthesis of nanomaterials for smart biopolymer packaging: challenges and outlooks. Journal of Nanostructure in Chemistry, 1-24.
105. Sumesh, K.R., Kanthavel, K. Green Synthesis of Aluminium Oxide Nanoparticles and its Applications in Mechanical and Thermal Stability of Hybrid Natural Composites. *J Polym Environ* **27**, 2189–2200 (2019). <https://doi.org/10.1007/s10924-019-01506-y>
106. Manikandan, V., Jayanthi, P., Priyadharsan, A., Vijayaprathap, E., Anbarasan, P., & Velmurugan, P. (2019). Green synthesis of pH-responsive Al2O3 nanoparticles: Application to rapid removal of nitrate ions with enhanced antibacterial activity. *Journal of Photochemistry and Photobiology A: Chemistry*, *371*, 205-215. https://doi.org/10.1016/j.jphotochem.2018.11.009
107. Gautam, Y. K., Sharma, K., Tyagi, S., Kumar, A., & Singh, B. P. (2022). Applications of green nanomaterials in coatings. Green Nanomaterials for Industrial Applications, 107-152. https://doi.org/10.1016/B978-0-12-823296-5.00014-9
108. Wang, H., Wick, R. L., & Xing, B. (2009). Toxicity of nanoparticulate and bulk ZnO, Al2O3 and TiO2 to the nematode Caenorhabditis elegans. Environmental Pollution, 157(4), 1171-1177.
109. Pattanayak, D., Pal, D., Thakur, C., Kumar, S., & Devnani, G. (2021). Bio-synthesis of iron nanoparticles for environmental remediation: Status till date. *Materials Today: Proceedings*, *44*, 3150-3155. <https://doi.org/10.1016/j.matpr.2021.02.821>
110. Le Wee, J., Law, M. C., Chan, Y. S., Choy, S. Y., & Tiong, A. N. T. (2022). The potential of Fe‐based magnetic nanomaterials for the agriculture sector. ChemistrySelect, 7(17), e202104603.
111. Mondal, P., Anweshan, A., & Purkait, M. K. (2020). Green synthesis and environmental application of iron-based nanomaterials and nanocomposite: A review. Chemosphere, 259, 127509. https://doi.org/10.1016/j.chemosphere.2020.127509
112. Kumar, A., Kuang, Y., Liang, Z., & Sun, X. (2020). Microwave chemistry, recent advancements, and eco-friendly microwave-assisted synthesis of nanoarchitectures and their applications: a review. Materials Today Nano, 11, 100076.
113. Gubala, V., Johnston, L. J., Liu, Z., Krug, H., Moore, C. J., Ober, C. K., ... & Vert, M. (2018). Engineered nanomaterials and human health: Part 1. Preparation, functionalization and characterization (IUPAC Technical Report). Pure and Applied Chemistry, 90(8), 1283-1324.
114. Sunny, N. E., Mathew, S. S., Chandel, N., Saravanan, P., Rajeshkannan, R., Rajasimman, M., Vasseghian, Y., Rajamohan, N., & Kumar, S. V. (2022). Green synthesis of titanium dioxide nanoparticles using plant biomass and their applications- A review. *Chemosphere*, *300*, 134612. <https://doi.org/10.1016/j.chemosphere.2022.134612>
115. Bekele, E. T., Gonfa, B. A., Zelekew, O. A., Belay, H. H., & Sabir, F. K. (2020). Synthesis of titanium oxide nanoparticles using root extract of Kniphofia foliosa as a template, characterization, and its application on drug resistance bacteria. Journal of Nanomaterials, 2020(1), 2817037.
116. Shiva Samhitha, S., Raghavendra, G., Quezada, C., & Hima Bindu, P. (2022). Green synthesized TiO2 nanoparticles for anticancer applications: Mini review. Materials Today: Proceedings, 54, 765-770. https://doi.org/10.1016/j.matpr.2021.11.073
117. Verma, V., Singh, J., Rawat, M., Kordy, M. G., & Shaban, M. (2022). A Review on Green Synthesis of TiO2 NPs: Photocatalysis and Antimicrobial Applications. Polymers, 14(7), 1444. https://doi.org/10.3390/polym14071444
118. Qidwai, A., Kumar, R., Shukla, S. K., & Dikshit, A. (2018). Advances in biogenic nanoparticles and the mechanisms of antimicrobial effects. Indian Journal of Pharmaceutical Sciences, 80(4), 592-603.
119. Abdelghany, T. M., Al-Rajhi, A. M. H., Al Abboud, M. A., Alawlaqi, M. M., Ganash, M., & Ben Rebah, F. (2018). Recent advances in green synthesis of silver nanoparticles and their applications: about future directions. A review. BioNanoScience, 8(1), 5–16. https://doi.org/10.1007/s12668-017-0413-9
120. Ahmed, S., Chaudhry, S. A., & Ikram, S. (2017). A review on biogenic synthesis of ZnO nanoparticles using plant extracts and microbes: A prospect towards green chemistry. Journal of Photochemistry and Photobiology B: Biology, 166, 272–284. <https://doi.org/10.1016/j.jphotobiol.2016.12.011>
121. Hano, C., & Abbasi, B. H. (2022). Plant-based green synthesis of nanoparticles: Production, characterization and applications. Biomolecules, 12(1), 31. <https://doi.org/10.3390/biom12010031>
122. Iravani, S. (2011). Green synthesis of metal nanoparticles using plants. Green Chemistry, 13(10), 2638–2650. https://doi.org/10.1039/C1GC15386B
123. Kuppusamy, P., Yusoff, M. M., Maniam, G. P., & Govindan, N. (2016). Biosynthesis of metallic nanoparticles using plant derivatives and their new avenues in pharmacological applications–An updated report. Saudi Pharmaceutical Journal, 24(4), 473–484. <https://doi.org/10.1016/j.jsps.2014.11.013>
124. Singh, P., Kim, Y. J., Zhang, D., & Yang, D. C. (2016). Biological synthesis of nanoparticles from plants and microorganisms. Trends in Biotechnology, 34(7), 588–599. <https://doi.org/10.1016/j.tibtech.2016.02.006>
125. Vigneshwaran, N., Kathe, A. A., Varadarajan, P. V., Nachane, R. P., & Balasubramanya, R. H. (2007). Silver–protein (core–shell) nanoparticle production using spent mushroom substrate. Langmuir, 23(13), 7113–7117. https://doi.org/10.1021/la063427s
126. Chandra, H., Patel, D., Kumari, P., Jangwan, J., & Yadav, S. (2019). Phyto-mediated synthesis of zinc oxide nanoparticles of Berberis aristata: Characterization, antioxidant activity and antibacterial activity with special reference to urinary tract pathogens. *Materials Science and Engineering: C*, *102*, 212-220. <https://doi.org/10.1016/j.msec.2019.04.035>
127. Barocio, E., Pipes, R. B., & Rodriguez, C. A. (2019). Characterization of the Mechanical Properties of FFF Structures and Materials: A Review on the Experimental, Computational and Theoretical Approaches. *Materials*, *12*(6), 895. <https://doi.org/10.3390/ma12060895>
128. Chandraker, S. K., Ghosh, M. K., Lal, M., & Shukla, R. (2021). A review on plant-mediated synthesis of silver nanoparticles, their characterization and applications. Nano Express, 2(2), 022008.
129. Ahamed, M., Majeed Khan, M., Siddiqui, M., AlSalhi, M. S., & Alrokayan, S. A. (2011). Green synthesis, characterization and evaluation of biocompatibility of silver nanoparticles. *Physica E: Low-dimensional Systems and Nanostructures*, *43*(6), 1266-1271. <https://doi.org/10.1016/j.physe.2011.02.014>
130. Poojary, M.M., Passamonti, P. & Adhikari, A.V. Green Synthesis of Silver and Gold Nanoparticles Using Root Bark Extract of Mammea suriga: Characterization, Process Optimization, and Their Antibacterial Activity. BioNanoSci. 6, 110–120 (2016). <https://doi.org/10.1007/s12668-016-0199-8>
131. Shen, X., Li, Z., Ma, L., Bian, X., Cheng, X., & Lou, X. (2022). Design and implementation of low-cost portable potentiostat based on WeChat. Journal of the Serbian Chemical Society, 87(5), 603-614.
132. Jung, H. S., Kim, K., & Kim, M. S. (1997). Raman spectroscopic investigation of the adsorption of 4-mercaptopyridine on a silver-sol surface. Journal of molecular structure, 407(2-3), 139-147.
133. Scotter, C. N. (1997). Non-destructive spectroscopic techniques for the measurement of food quality. Trends in food science & technology, 8(9), 285-292.
134. Kang, C. H., Dursun, I., Liu, G., Sinatra, L., Sun, X., Kong, M., ...& Ooi, B. S. (2019). High-speed colour-converting photodetector with all-inorganic CsPbBr3 perovskite nanocrystals for ultraviolet light communication. *Light: Science & Applications*, *8*(1), 94.
135. Singh Jassal, P., Kaur, D., Prasad, R., & Singh, J. (2022). Green synthesis of titanium dioxide nanoparticles: Development and applications. *Journal of Agriculture and Food Research*, *10*, 100361. <https://doi.org/10.1016/j.jafr.2022.100361>
136. Taha, M., Hassan, M., Essa, S., & Tartor, Y. (2013). Use of Fourier transform infrared spectroscopy (FTIR) spectroscopy for rapid and accurate identification of Yeasts isolated from human and animals. International journal of veterinary science and medicine, 1(1), 15-20.
137. Alburae, N., Alshamrani, R., & Mohammed, A. E. (2024). Bioactive silver nanoparticles fabricated using Lasiurus scindicus and Panicum turgidum seed extracts: Anticancer and antibacterial efficiency. Scientific Reports, 14(1), 4162.
138. Nedoma, J., Zboril, O., Fajkus, M., Cubik, J., Zavodny, P., Novak, M., ... & Vasinek, V. (2016, April). Fiber optic interferometer as a security element. In Optical Sensing and Detection IV (Vol. 9899, pp. 673-681). SPIE.
139. Mohamed, M., Jaafar, J., Ismail, A., Othman, M., & Rahman, M. (2017). Fourier Transform Infrared (FTIR) Spectroscopy. *Membrane Characterization*, 3-29. <https://doi.org/10.1016/B978-0-444-63776-5.00001-2>
140. Fisicaro, M., Steenbergen, T. A., Doedes, Y. C., Heeck, K., & Löffler, W. (2025). Imaging transverse modes in a gigahertz surface-acoustic-wave cavity. Physical Review Applied, 23(1), 014032.
141. Soumyananda, C., Shamila, S., & Pinak, C. (2013). The Effect of the Binding of ZnO Nanoparticle on the Structure and Stability of α-Lactalbumin: A Comparative Study.
142. Takahashi, Y., Zettsu, N., Nishino, Y., Tsutsumi, R., Matsubara, E., Ishikawa, T., & Yamauchi, K. (2010). Three-dimensional electron density mapping of shape-controlled nanoparticle by focused hard X-ray diffraction microscopy. *Nano letters*, *10*(5), 1922-1926.
143. Whitlock, R. R. (2000). *Transmission Cathode of X Ray Production*. DEPARTMENT OF THE NAVY WASHINGTON DC.
144. Frauenfelder, H., & Frauenfelder, H. (2010). Scattering of Photons: X-Ray Diffraction. *The Physics of Proteins: An Introduction to Biological Physics and Molecular Biophysics*, 341-361.
145. Bragg W L 1913 Containing papers of a mathematical and physical character 89 248–77
146. Sadeghi, B., & Gholamhoseinpoor, F. (2015). A study on the stability and green synthesis of silver nanoparticles using Ziziphora tenuior (Zt) extract at room temperature. *Spectrochimica Acta Part A: Molecular and Biomolecular Spectroscopy*, *134*, 310-315.
147. Holder, C. F., & Schaak, R. E. (2019). Tutorial on powder X-ray diffraction for characterizing nanoscale materials. *Acs Nano*, *13*(7), 7359-7365.
148. Gumustas, M., Sengel-Turk, C. T., Gumustas, A., Ozkan, S. A., & Uslu, B. (2017). Effect of Polymer-Based Nanoparticles on the Assay of Antimicrobial Drug Delivery Systems. *Multifunctional Systems for Combined Delivery, Biosensing and Diagnostics*, 67-108. <https://doi.org/10.1016/B978-0-323-52725-5.00005-8>
149. Filipponi, L., & Sutherland, D. (2010). **Chapter 6 - Characterisation methods.** In NANOYOU Teachers Training Kit in Nanotechnologies (Vol. January, pp. 1–13).
150. Johal, M. (2011). Understanding nanomaterials. CRC Press. <https://www.crcpress.com/UnderstandingNanomaterials/Johal/p/book/9781138441569>
151. Juan, R., Wang, M., Lian, J., Gu, C., Li, L., & Bao, Y. (2021). Quantifying the Comprehensive Characteristics of Inclusion-Induced Defects Using an Integrated Destructive and Non-Destructive Method. Materials, 14(6), 1475.
152. Zaefferer, S., & Elhami, N. N. (2014). Theory and application of electron channelling contrast imaging under controlled diffraction conditions. *Acta Materialia*, *75*, 20-50.
153. Etefagh, R., Azhir, E., & Shahtahmasebi, N. (2013). Synthesis of CuO nanoparticles and fabrication of nanostructural layer biosensors for detecting Aspergillus niger fungi. *Scientia Iranica*, *20*(3), 1055-1058.
154. Bradbury, S. , Joy, . David C. and Ford, . Brian J. (2023, March 20). *scanning electron microscope*. *Encyclopedia Britannica*. <https://www.britannica.com/technology/scanning-electron-microscope>
155. Raza, M. A., Kanwal, Z., Rauf, A., Sabri, A. N., Riaz, S., & Naseem, S. (2016). Size-and shape-dependent antibacterial studies of silver nanoparticles synthesized by wet chemical routes. *Nanomaterials*, *6*(4), 74.
156. Inkson, B. (2016). Scanning electron microscopy (SEM) and transmission electron microscopy (TEM) for materials characterization. *Materials Characterization Using Nondestructive Evaluation (NDE) Methods*, 17-43. <https://doi.org/10.1016/B978-0-08-100040-3.00002-X>
157. Fultz B and Howe J M 2012 Transmission electron microscopy and diffractometry of materials (Berlin: Springer) (https://doi.org/ 10.1007/978-3-642-29761-8)
158. Avadhani G S 2010 Techniques for characterization of nano materials in Proceedings of the Sixth International Conference on Mathematical Modeling and Computer Simulation of Material Technologies, (MMT-2010) (Ariel, Israel)
159. Harrison, C., Park, M., Chaikin, P. M., Register, R. A., Adamson, D. H., & Yao, N. (1998). Layer by layer imaging of diblock copolymer films with a scanning electron microscope. *Polymer*, *39*(13), 2733-2744.
160. Yu, K., Lu, G., Bo, Z., Mao, S., & Chen, J. (2011). Carbon nanotube with chemically bonded graphene leaves for electronic and optoelectronic applications. *The Journal of Physical Chemistry Letters*, *2*(13), 1556-1562.
161. Ford, B. J. , Joy, . David C. and Bradbury, . Savile (2023, March 20). transmission electron microscope. Encyclopedia Britannica. <https://www.britannica.com/technology/transmission-electron-microscope>
162. Begum, S. J., Pratibha, S., Rawat, J. M., Venugopal, D., Sahu, P., Gowda, A., Qureshi, K. A., & Jaremko, M. (2022). Recent Advances in Green Synthesis, Characterization, and Applications of Bioactive Metallic Nanoparticles. *Pharmaceuticals*, *15*(4), 455. <https://doi.org/10.3390/ph15040455>
163. Kalimuthu, K., Cha, B. S., Kim, S., & Park, K. S. (2019). Eco-friendly synthesis and biomedical applications of gold nanoparticles: A review. Microchemical Journal, 152, 104296. https://doi.org/10.1016/j.microc.2019.104296
164. Jadoun, S., Arif, R., Jangid, N. K., & Meena, R. K. (2020). Green synthesis of nanoparticles using plant extracts: A review. Environmental Chemistry Letters, 19, 355–374. https://doi.org/10.1007/s10311-020-01094-8
165. Luna, C., Chávez, V., Barriga-Castro, E. D., Núñez, N. O., & Mendoza-Reséndez, R. (2015). Biosynthesis of silver fine particles and particles decorated with nanoparticles using the extract of Illicium verum (star anise) seeds. Spectrochimica Acta Part A: Molecular and Biomolecular Spectroscopy, 141, 43–50. <https://doi.org/10.1016/j.saa.2015.01.050>
166. Bhattacharjee, S. (2016). DLS and zeta potential – What they are and what they are not? Journal of Controlled Release, 235, 337–351. https://doi.org/10.1016/j.jconrel.2016.06.017
167. Patil, S., & Chandrasekaran, R. (2020). Biogenic nanoparticles: A comprehensive perspective in synthesis, characterization, application and its challenges. Journal of Genetic Engineering and Biotechnology, 18(1), 1-23. <https://doi.org/10.1186/s43141-020-00081-3>
168. Amooaghaie, R., Saeri, M. R., & Azizi, M. (2015). Synthesis, characterization and biocompatibility of silver nanoparticles synthesized from Nigella sativa leaf extract in comparison with chemical silver nanoparticles. Ecotoxicology and Environmental Safety, 120, 400–408. <https://doi.org/10.1016/j.ecoenv.2015.06.025>
169. Singh, A. K., Pal, P., Gupta, V., et al. (2018). Green synthesis, characterization and antimicrobial activity of zinc oxide quantum dots using Eclipta alba. Materials Chemistry and Physics, 203, 40–48. https://doi.org/10.1016/j.matchemphys.2017.09.049
170. Bhattacharya, R., & Mukherjee, P. (2008). Biological properties of "naked" metal nanoparticles. Advanced Drug Delivery Reviews, 60(11), 1289–1306. https://doi.org/10.1016/j.addr.2008.03.013
171. Mironava, T., Hadjiargyrou, M., Simon, M., et al. (2010). Gold nanoparticles cellular toxicity and recovery: effect of size, concentration and exposure time. Nanotoxicology, 4(1), 120–137. <https://doi.org/10.3109/17435390903418993>
172. Gadad, A. P., Vijay Kumar, S. V., Dandagi, P. M., Bolmol, U. B., & Pavani, N. (2014). Formulation and evaluation of chitosan nanoparticles containing lamivudine. International Journal of Pharmaceutical Sciences and Research, 5(5), 1790–1795.​
173. Mittal, J., Kaur, G., Nayak, V., & Sharma, S. (2014). Nanotechnology in medicine and antibacterial effect of silver nanoparticles. Advances in Natural Sciences: Nanoscience and Nanotechnology, 5(4), 043002. <https://doi.org/10.1088/2043-6262/5/4/043002>​
174. Gómez-Romero, P. (2001). Hybrid organic–inorganic materials—in search of synergic activity. Advanced Materials, 13(3), 163–174. <https://doi.org/10.1002/1521-4095(200102)13:3><163::AID-ADMA163>3.0.CO;2-M​
175. Qiu, H., Rieger, B., Gilbert, R. G., Jérôme, R., & Jérôme, C. (2004). Synthesis of well-defined poly(ε-caprolactone)-block-poly(N-isopropylacrylamide) copolymers and their micellization in water. Chemistry of Materials, 16(5), 850–856. <https://doi.org/10.1021/cm030602e>​
176. AshaRani, P. V., Mun, G. L. K., Hande, M. P., & Valiyaveettil, S. (2009). Cytotoxicity and genotoxicity of silver nanoparticles in human cells. ACS Nano, 3(2), 279–290. <https://doi.org/10.1021/nn800547q>​
177. Li, W. R., Xie, X. B., Shi, Q. S., Duan, S. S., Ouyang, Y. S., & Chen, Y. B. (2011). Antibacterial effect of silver nanoparticles on Staphylococcus aureus. Biometals, 24(1), 135–141. <https://doi.org/10.1007/s10534-010-9381-0>​
178. Pollini, M., Paladini, F., Catalano, M., Taurino, A., Licciulli, A., Maffezzoli, A., & Sannino, A. (2011). Antibacterial coatings on textiles: The influence of surface roughness on bacteria adhesion. Journal of Materials Science: Materials in Medicine, 22(9), 2005–2012. <https://doi.org/10.1007/s10856-011-4395-6>
179. Jasmine, Singh, N., Nagpal, D., Puniani, S., & Gupta, P. (2025). Golden Therapeutic Approach to Combat Viral Diseases Using Gold Nanomaterials. ASSAY and Drug Development Technologies, 23(2), 70-83.
180. Darouiche, R. O., Raad, I. I., Heard, S. O., Thornby, J. I., Wenker, O. C., Gabrielli, A., & Harris, R. L. (1999). A comparison of two antimicrobial-impregnated central venous catheters. The New England Journal of Medicine, 340(1), 1–8. https://doi.org/10.1056/NEJM199901073400101
181. Leaper, D. J. (2006). Silver dressings: Their role in wound management. International Wound Journal, 3(4), 282–294. https://doi.org/10.1111/j.1742-481X.2006.00265.x
182. Ren, G., Hu, D., Cheng, E. W., Vargas-Reus, M. A., Reip, P., & Allaker, R. P. (2009). Characterisation of copper oxide nanoparticles for antimicrobial applications. International Journal of Antimicrobial Agents, 33(6), 587–590. https://doi.org/10.1016/j.ijantimicag.2008.12.004
183. Dobrucka, R. (2019). Selected applications of metal nanoparticles in medicine and pharmacology. LogForum, 15(4), 449-457.
184. Qi, B., Wang, C., Ding, J., & Tao, W. (2019). Editorial: Applications of Nanobiotechnology in Pharmacology. *Frontiers in Pharmacology*, *10*. <https://doi.org/10.3389/fphar.2019.01451>
185. Mohammed, A. E., et al. (2018). Antibacterial and cytotoxic potential of biosynthesized silver nanoparticles by some plant extracts. Nanomaterials, 8(6), 382. https://doi.org/10.3390/nano8060382
186. Poopathi, S., De Britto, L. J., & Ragavendran, C. (2015). Synthesis of silver nanoparticles from Azadirachta indica – a most effective method for mosquito control. Environmental Science and Pollution Research, 22(4), 2956–2963.
187. Amooaghaie, R., Saeri, M. R., & Azizi, M. (2015). Synthesis, characterization and biocompatibility of silver nanoparticles synthesized from Nigella sativa leaf extract in comparison with chemical silver nanoparticles. Ecotoxicology and Environmental Safety, 120, 400–408.
188. Rasmussen, J. W., et al. (2010). Zinc oxide nanoparticles for selective destruction of tumor cells and potential for drug delivery applications. Expert Opinion on Drug Delivery, 7(9), 1063–1077.
189. Du, J., Singh, H., & Yi, T. H. (2016). Antibacterial, anti-biofilm and anticancer potentials of green synthesized silver nanoparticles using benzoin gum (Styrax benzoin) extract. Bioprocess and Biosystems Engineering, 39(12), 1923–1931.
190. Park, Y., et al. (2011). Polysaccharides and phytochemicals: a natural reservoir for the green synthesis of gold and silver nanoparticles. IET Nanobiotechnology, 5(3), 69–78.
191. Singh, P., et al. (2016). Biological synthesis of nanoparticles from plants and microorganisms. Trends in Biotechnology, 34(7), 588–599.
192. Kuppusamy, P., et al. (2016). Biosynthesis of metallic nanoparticles using plant derivatives and their new avenues in pharmacological applications – An updated report. Saudi Pharmaceutical Journal, 24(4), 473–484.
193. Prateek Mathur, et al. (2018). Pharmaceutical aspects of silver nanoparticles. Artificial Cells, Nanomedicine, and Biotechnology, 46(sup1), 115–126. https://doi.org/10.1080/21691401.2017.1414825
194. Gunti, L., Dass, R. S., & Kalagatur, N. K. (2019). Phytofabrication of Selenium Nanoparticles from Emblica officinalis Fruit Extract and Exploring Its Biopotential Applications. Frontiers in Microbiology, 10, 931. <https://doi.org/10.3389/fmicb.2019.00931>
195. Ahmed, M. (2020). Nanomaterial synthesis. In Polymer Science and Nanotechnology (pp. 361–399). <https://doi.org/10.1016/B978-0-12-816806-6.00016-9>
196. Ahmed, S., Chaudhry, S. A., & Ikram, S. (2017). A review on biogenic synthesis of ZnO nanoparticles using plant extracts and microbes: A prospect towards green chemistry. Journal of Photochemistry and Photobiology B: Biology, 166, 272–284. <https://doi.org/10.1016/j.jphotobiol.2016.12.011>
197. Kumar, S., Basumatary, I. B., Sudhani, H. P., Bajpai, V. K., Chen, L., Shukla, S., & Mukherjee, A. (2021). Plant extract mediated silver nanoparticles and their applications as antimicrobials and in sustainable food packaging: A state-of-the-art review. Trends in Food Science & Technology, 112, 651–666. <https://doi.org/10.1016/j.tifs.2021.04.031>
198. Poopathi, S., De Britto, L. J., & Ragavendran, C. (2015). Synthesis of silver nanoparticles from Azadirachta indica – a most effective method for mosquito control. Environmental Science and Pollution Research International, 22(4), 2956–2963. <https://doi.org/10.1007/s11356-014-3487-4>
199. Zahir, A. A., Bagavan, A., Kamaraj, C., Elango, G., & Rahuman, A. A. (2015). Green synthesis of silver and titanium dioxide nanoparticles using Euphorbia prostrata extract shows shift from apoptosis to G0/G1 arrest followed by necrotic cell death in Leishmania donovani. Antimicrobial Agents and Chemotherapy, 59(8), 4782–4799. <https://doi.org/10.1128/AAC.00111-15>
200. Basova, T. V., Vikulova, E. S., Dorovskikh, S. I., Hassan, A., & Morozova, N. B. (2021). The use of noble metal coatings and nanoparticles for the modification of medical implant materials. *Materials & Design*, *204*, 109672. <https://doi.org/10.1016/j.matdes.2021.109672>
201. Yun’an Qing, Lin Cheng, Ruiyan Li, Guancong Liu, Yanbo Zhang, Xiongfeng Tang, Jincheng Wang, He Liu & Yanguo Qin (2018) Potential antibacterial mechanism of silver nanoparticles and the optimization of orthopedic implants by advanced modification technologies, International Journal of Nanomedicine, 13:, 3311-3327, DOI: [10.2147/IJN.S165125](https://doi.org/10.2147/IJN.S165125)
202. Datte, C. E., Nishioka, R. S., Bottino, M. A., Evangelhista, A. D. M., de M Monteiro, F. M., & Borges, A. L. S. (2018). Influence of different restorative materials on the stress distribution in dental implants. Journal of clinical and experimental dentistry, 10(5), e439.
203. Canullo, L., Rosa, J. C., Pinto, V. S., Francischone, C. E., & Götz, W. (2012). Inward-inclined implant platform for the amplified platform-switching concept: 18-month follow-up report of a prospective randomized matched-pair controlled trial. International Journal of Oral & Maxillofacial Implants, 27(4), 927–934.
204. Simonis, P., Dufour, T., & Tenenbaum, H. (2010). Long-term implant survival and success: A 10–16-year follow-up of non-submerged dental implants. Clinical Oral Implants Research, 21(7), 772–777. <https://doi.org/10.1111/j.1600-0501.2010.01912.x>
205. Frost, H. M. (2004). A 2003 update of bone physiology and Wolff’s Law for clinicians. The Angle Orthodontist, 74(1), 3–15. https://doi.org/10.1043/0003-3219(2004)074<0003:AUOBPA>2.0.CO;2
206. Brunette, D. M., Tengvall, P., Textor, M., & Thomsen, P. (Eds.). (2001). Titanium in medicine. Springer. https://doi.org/10.1007/978-3-642-56486-4
207. Mahabir, R. C., & Butler, C. E. (2011, February). Stabilization of the chest wall: autologous and alloplastic reconstructions. In Seminars in plastic surgery (Vol. 25, No. 01, pp. 034-042). © Thieme Medical Publishers.
208. Ayawanna, J., Kingnoi, N., & Laorodphan, N. (2019). A feasibility study of egg shell-derived porous glass–ceramic orbital implants. Materials Letters, 241, 39-42.
209. Chen, Q., & Thouas, G. A. (2015). Metallic implant biomaterials. *Materials Science and Engineering: R: Reports*, *87*, 1-57.
210. Kirilova, I. A., Sadovoy, M. A., Podorozhnaya, V. T., Buyakova, S. P., & Kulkov, S. N. (2013). Ceramic and osteoceramic implants: upcoming trends. Russian Journal of Spine Surgery (Khirurgiya Pozvonochnika), (4), 052-062.
211. M. Andreiotelli, H.J. Wenz and R.-J. KohalClin. Oral Implants Res., 20 (Suppl 4) (2009), pp. 32-47, [10.1111/j.1600-0501.2009.01785.x](https://doi.org/10.1111/j.1600-0501.2009.01785.x)
212. H.J. Conrad, W.-J. Seong and I.J. PesunJ. Prosthet. Dent., 98 (2007), pp. 389-404, [10.1016/S0022-3913(07)60124-3](https://doi.org/10.1016/S0022-3913(07)60124-3)
213. S.V. DorozhkinBiomaterials., 31 (2010), pp. 1465-1485, [10.1016/j.biomaterials.2009.11.050](https://doi.org/10.1016/j.biomaterials.2009.11.050)
214. L.L. HenchJ. Mater. Sci. Mater. Med., 26 (2015), p. 86, [10.1007/s10856-015-5425-3](https://doi.org/10.1007/s10856-015-5425-3)
215. P. De Aza, A. De Aza and S. De AzaBol Soc. Esp. Ceram Vidr., 44 (2006), pp. 135-145
216. S. Madeira, … +4 … , O. CarvalhoCeram. Int., 46 (2020), pp. 7109-7121, [10.1016/j.ceramint.2019.11.203](https://doi.org/10.1016/j.ceramint.2019.11.203)
217. Sandoval-Yañez, C., Escobar, L., & Amador, C. A. (2020). The advantages of polymeric hydrogels in calcineurin inhibitor delivery. *Processes*, *8*(11), 1331.
218. Ku, S. H., Lee, M., & Park, C. B. (2013). Carbon‐based nanomaterials for tissue engineering. Advanced healthcare materials, 2(2), 244-260.
219. Jalilinejad, N., Rabiee, M., Baheiraei, N., Ghahremanzadeh, R., Salarian, R., Rabiee, N., ... & Zare, E. N. (2023). Electrically conductive carbon‐based (bio)‐nanomaterials for cardiac tissue engineering. Bioengineering & Translational Medicine, 8(1), e10347.
220. Samuel, M. S., Ravikumar, M., John J, A., Selvarajan, E., Patel, H., Chander, P. S., ... & Chandrasekar, N. (2022). A review on green synthesis of nanoparticles and their diverse biomedical and environmental applications. Catalysts, 12(5), 459.
221. Hirche, T. O., Knoop, C., Hebestreit, H., Shimmin, D., Solé, A., Elborn, J. S., ... & Ecorn-Cf Study Group. (2014). Practical guidelines: lung transplantation in patients with cystic fibrosis. Pulmonary medicine, 2014(1), 621342.
222. Ume, A. C., Pugh, J. M., Kemp, M. G., & Williams, C. R. (2020). Calcineurin inhibitor (CNI)‐associated skin cancers: New insights on exploring mechanisms by which CNIs downregulate DNA repair machinery. *Photodermatology, photoimmunology & photomedicine*, *36*(6), 433-440.
223. Zarenezhad, E., Hadi, A. T., Nournia, E., Rostamnia, S., & Ghasemian, A. (2023). A Comprehensive Review on Potential In Silico Screened Herbal Bioactive Compounds and Host Targets in the Cardiovascular Disease Therapy. *BioMed Research International*, *2024*(1), 2023620. <https://doi.org/10.1155/2024/2023620>
224. Soliman, M.K.Y., Salem, S.S., Abu-Elghait, M. *et al.* Biosynthesis of Silver and Gold Nanoparticles and Their Efficacy Towards Antibacterial, Antibiofilm, Cytotoxicity, and Antioxidant Activities. *Appl Biochem Biotechnol* **195**, 1158–1183 (2023). https://doi.org/10.1007/s12010-022-04199-7
225. Schaad, N. W., & Frederick, R. D. (2002). Real-time PCR and its application for rapid plant disease diagnostics. *Canadian Journal of Plant Pathology, 24*, 250–258.
226. Ahmed, S., Chaudhry, S. A., & Ikram, S. (2017). A review on biogenic synthesis of ZnO nanoparticles using plant extracts and microbes: A prospect towards green chemistry. Journal of Photochemistry and Photobiology B: Biology, 166, 272–284. <https://doi.org/10.1016/j.jphotobiol.2016.12.011>
227. Boenink, M., Swierstra, T., & Stemerding, D. (2010). Anticipating the interaction between technology and morality: A scenario study of experimenting with humans in bionanotechnology. Studies in Ethics, Law and Technology, 4(2). https://doi.org/10.2202/1941-6008.1110
228. Gunti, L., Dass, R. S., & Kalagatur, N. K. (2019). Phytofabrication of selenium nanoparticles from Emblica officinalis fruit extract and exploring its biopotential applications: Antioxidant, antimicrobial, and biocompatibility. Frontiers in Microbiology, 10, 931. <https://doi.org/10.3389/fmicb.2019.00931>
229. Hano, C., & Abbasi, B. H. (2022). Plant-based green synthesis of nanoparticles: Production, characterization and applications. Biomolecules, 12(1), 31. <https://doi.org/10.3390/biom12010031>
230. Kuppusamy, P., Yusoff, M. M., Maniam, G. P., & Govindan, N. (2016). Biosynthesis of metallic nanoparticles using plant derivatives and their new avenues in pharmacological applications – An updated report. Saudi Pharmaceutical Journal, 24(4), 473–484. <https://doi.org/10.1016/j.jsps.2014.11.013>
231. Singh, P., Kim, Y., Zhang, D., & Yang, D. (2016). Biological synthesis of nanoparticles from plants and microorganisms. Trends in Biotechnology, 34(7), 588–599. <https://doi.org/10.1016/j.tibtech.2016.02.006>
232. Mohammed, A. E., Al-Qahtani, A., Al-Mutairi, A., Al-Shamri, B., & Aabed, K. (2018). Antibacterial and cytotoxic potential of biosynthesized silver nanoparticles by some plant extracts. Nanomaterials, 8(6), 382. https://doi.org/10.3390/nano8060382
233. Jenkins, M. J., & Farid, S. S. (2015). Human pluripotent stem cell‐derived products: advances towards robust, scalable and cost‐effective manufacturing strategies. *Biotechnology journal*, *10*(1), 83-95.
234. Patil, S., & Chandrasekaran, R. (2020). Biogenic nanoparticles: A comprehensive perspective in synthesis, characterization, application and its challenges. Journal of Genetic Engineering and Biotechnology, 18(1), 1–23. <https://doi.org/10.1186/s43141-020-00081-3>
235. Kumar, S., et al. (2023). Advantages and disadvantages of metal nanoparticles. In Nanoparticles Reinforced Metal Nanocomposites (pp. 209–235). Springer Nature Singapore. https://doi.org/10.1007/978-981-19-7801-5_11
236. Jeliazkova, N., Longhin, E., El Yamani, N., Rundén-Pran, E., Moschini, E., Serchi, T., ... & Dusinska, M. (2024). A template wizard for the cocreation of machine-readable data-reporting to harmonize the evaluation of (nano) materials. *Nature protocols*, *19*(9), 2642-2684.
